# Transcriptomic profiling of *Methylococcus capsulatus* (Bath) during growth with two different methane monooxygenases

**DOI:** 10.1002/mbo3.324

**Published:** 2015-12-20

**Authors:** Øivind Larsen, Odd A. Karlsen

**Affiliations:** ^1^ Uni Research EnvironmentThormøhlensgate 49bBergen5006Norway; ^2^ Department of Molecular BiologyUniversity of BergenBergenNorway

**Keywords:** Copper, *c*‐type cytochromes, methanotroph, microarray, respiration chain

## Abstract

*Methylococcus capsulatus* (Bath) is a methanotroph that possesses both a membrane‐embedded (pMMO) and a soluble methane monooxygenase (sMMO). The expression of these two MMO's is tightly controlled by the availability of copper in the growth medium, but the underlying mechanisms and the number of genes involved in this switch in methane oxidation is not yet fully elucidated. Microarray analyses were used to assess the transcriptome in cells producing either pMMO or sMMO. A total of 137 genes were differentially expressed, with 87 genes showing a significant up‐regulation during sMMO production. The majority of the differentially expressed genes could be assigned to functional roles in the energy metabolism and transport. Furthermore, three copper responding gene clusters were discovered, including an extended cluster that also harbors the genes for sMMO. Our data also indicates that major changes takes place in the respiratory chain between pMMO‐ and sMMO‐producing cells, and that quinone are predominantly used as the electron donors for methane oxidation by pMMO. Intriguingly, a large proportion of the differentially expressed genes between pMMO‐ and sMMO‐producing cells encode *c*‐type cytochromes. By combining microarray‐ and mass spectrometry data, a total of 35 *c*‐type cytochromes are apparently expressed in *M. capsulatus* when grown in nitrate mineral salt medium with methane as sole energy and carbon source, and the expression of 21 of these respond to the availability of copper. Interestingly, several of these *c*‐type cytochromes are recovered from the cell surface, suggesting that extracellular electron transfers may occur in *M*. *capsulatus*.

## Introduction

*Methylococcus capsulatus* (Bath) is an obligate aerobic methanotroph that utilize methane or methanol as sole carbon and energy source (Anthony 1982). It belongs to the gamma‐proteobacteria and is similar to type I methanotrophs by having an incomplete TCA cycle and stacks of internal membranes where also the enzyme for methane oxidation, the particulate methane monooxygenase (pMMO), is located (Hanson and Hanson 1996; Semrau et al. 2010). pMMO is a copper‐containing enzyme, and is dependent on both Cu(I) and Cu(II) for its catalytic activity (Lieberman and Rosenzweig 2005). However, under copper‐limited growth, the internal membranes disappear and an alternative soluble and cytoplasmic methane monooxygenase (sMMO) is expressed (Prior and Dalton 1985). In contrast to pMMO, sMMO is an Fe‐containing enzyme, and is not dependent on copper for methane oxidation (for a review on sMMO, see Merkx et al. 2001). This change in how methane is oxidized, in addition to the morphological changes that occurs in response to the bioavailability of copper, are known as the “copper switch”, and has gained considerable interest (Murrell et al. 2000; Hakemian and Rosenzweig 2007; Semrau et al. 2010). However, the underlying mechanisms and the number of genes involved in the “copper switch” are not yet fully elucidated.

*M. capsulatus* has been studied as a model organism for methanotrophy and was also the first methanotroph to have its genome fully sequenced (Ward et al. 2004). The genome sequence revealed a 3.3 Mb genome highly specialized for a methanotrophic lifestyle. It was also evident that there were a large proportion of duplicated genes and the possibility of redundant pathways encoded in the genome. One of the more interesting findings from the genome sequence was the presence of a high number of ORFs encoding putative *c*‐type cytochromes, a trait that is more common in bacteria with diverse metabolic and respiratory capabilities (Ward et al. 2004; Bertini et al. 2006). *c*‐type cytochromes are hemoproteins that are characterized by the covalent attachment of one or several heme groups. It is well known that *c*‐type cytochromes play an important role in methylotrophy by being the electron acceptor for methanol dehydrogenase, the second step in methane oxidation (Anthony 1986). Soluble *c*‐type cytochromes are present in high concentrations in *M. capsulatus* during methane oxidation, and have previously been grouped into four fractions after ammonium sulfate precipitation and gel filtration (Ambler et al. 1986; Zahn et al. 1994). During methanotrophic growth, *M. capsulatus* cells also express several multiheme cytochromes on the cell surface (Karlsen et al. 2005, 2008). The presence of multiheme *c*‐type cytochromes on the surface of *M. capsulatus* is intriguing. Surface exposed *c*‐type cytochromes are intimately linked to extracellular dissimilatory reduction of metals (Lovley 1993; Shi et al. 2007; Carlson et al. 2012), an ability not reported for *M*. *capsulatus* or any other methanotroph.

In order to further enhance our understanding of the “copper switch”, we have used microarray analyses to map the transcriptome in *M. capsulatus* during aerobic methane oxidation with either pMMO or sMMO. A total of 139 genes were differentially expressed between sMMO‐ and pMMO‐producing cells. The majority of the differentially expressed genes have a predictable functional role in energy and transport metabolisms. A more detailed interpretation of these data suggests potential changes in the composition of the respiratory chain when methane oxidation switches from pMMO to sMMO, and we present here a model suggesting how this change may occur. Most interestingly, the major functional category of differentially expressed genes included 21 *c*‐type cytochromes. Several of these are part of large gene clusters that respond strongly to the availability of copper. Among the 51 *c*‐type cytochromes present in the *M. capsulatus* genome, which includes four novel genome annotations from this study, we confirmed the expression of 24 of these at the protein level using LTQ‐ORBITRAP mass spectrometry.

## Experimental Procedures

### Growth of *M. capsulatus* bath

*Methylococcus capsulatus* Bath (NCIMB 11132) was grown in triplicates at 45°C in 100 mL batch cultures in nitrate mineral salt medium (NMS) (Whittenbury et al. 1970). CH_4_ and CO_2_ were added to a final atmosphere of 23% CH_4_, 6% CO_2_, and 18% O_2_. The cultures were grown without added CuSO_4_ (~0 *μ*mol/L copper) or with copper added to the medium to a final concentration of 0.8, 1.6, and 5.0 *μ*mol/L CuSO_4_. Cultures were screened for sMMO activity by the naphthalene assay (Brusseau et al. 1990). The cultures were grown to an optical density between 0.6 and 0.8. For RNA analysis, two times 10 mL culture was taken out and mixed with RNAlater (Qiagen, Valencia, CA) and treated in accordance with the manufacturer's recommendations. The remaining cultures were divided into two, harvested by centrifugation at 5000× ***g*** for 20 min, and stored cold before subcellular fractionation.

### Subcellular fractionation

Surface‐associated proteins were extracted as previously described (Karlsen et al. 2005). Periplasmic proteins were extracted by the osmotic shock method (Neu and Heppel 1965). Inner and outer membrane fractions were obtained as described by Fjellbirkeland et al. (1997).

### Total RNA isolation, purification, RT‐PCR, and quantitative RT‐PCR

Total RNA was extracted using RNeasy^®^ Plus Mini kit (Qiagen). Contaminating DNA was removed by treatment with 2 U of RNase‐free DNase I (NEB) for 15 min at 37°C, followed by RNeasy^®^ MinElute^™^ Cleanup kit (Qiagen). The RNA was eluted in 20 *μ*L RNase‐free water. The amount of RNA was quantified using a NanoDrop 1000 spectrophotometer (Thermo Scientific, Waltham, MA), and its integrity was checked on a 2100 BioAnalyzer (Agilent Technologies, Santa Clara, CA). The absence of contaminating DNA was verified with RT‐PCR by omitting the reverse transcriptase step using primers targeting the *mopB* gene (Table S3).

First‐strand cDNA synthesis for qPCR was performed on 500 ng total RNA, using 250 U of Transcriptor reverse transcriptase (Roche, Oslo, Norway) and random hexamer at 42°C for 30 min. cDNA (approximately 10 ng RNA) was amplified in 20 *μ*L reaction volume that contained 1× LightCycler^®^ 480 SYBR Green I master mix (Roche) and with the primers listed in Table S3. The reactions were performed in triplicate on two independent sample sets (0, 0.8, 1.6, and 5.0 *μ*mol/L CuSO_4_ added to the growth medium) using the following cycling parameters: one cycle of 10 min at 95°C followed by 45 cycles of 10 sec at 95°C, 10 sec at 55°C, and 10 sec at 72°C. The transcription level of specific genes were normalized to the transcription of *mopB,* which previously has been used as a housekeeping gene in qPCR analyses of *M. capsulatus* (Karlsen et al. 2005). The results were interpreted using the 2^−ΔΔCt^ calculation method (Livak and Schmittgen 2001) on samples with Ct < 40 cycles.

### Transcriptome analysis using *M. capsulatus* DNA microarrays

A custom‐made Gene Expression microarray was ordered from Roche Nimblegen where each gene was covered by eight probes per sequence and with five replicates on each microarray. A random oligomer with no match in the genome of *M. capsulatus* was used as a negative probe. A total of 3007 exemplars for 3041 ORF's were spotted on the microarray. Total RNA from three biological replicates from each growth condition (0, 0.8, 1.6, and 5.0 *μ*mol/L CuSO_4_ added to the growth medium) was sent to Roche Nimblegen for cDNA synthesis, labeling, and hybridization. Normalized data were analyzed using the J‐Express software package (Dysvik and Jonassen 2001). ANOVA statistics were used to find statistically significant differentially expressed transcripts between the culture conditions. A *P*‐value below 0.02 was considered significant.

### Heme staining

Proteins separated by SDS‐PAGE were transferred to a nitrocellulose membrane by electroblotting and stained for *c*‐type heme peroxidase activity using enhanced chemiluminescence (ECL) (Dorward 1993; Vargas et al. 1993).

### Spectrophotometric analyses

Spectrophotometric absorption data were obtained using 1‐cm path length quartz cuvettes in a UNICAM UV/VIS UV2 spectrophotometer. Reduced‐minus‐oxidized absorbance spectra were recorded by adding 5 *μ*L of 20% H_2_O_2_ to the sample contained in the reference cuvette and a few crystals of dithionite to the sample cuvette as described by Niederman (1974).

### Mass spectrometry analyses

Sample preparation and LTQ‐OBITRAP mass spectrometry analyses of cellular fractions were performed by the mass spectrometry service facility at the University of Oslo, Norway. Mascot (Matrix Science, London, UK) software was used for database searches toward the in‐house *M. capsulatus* database (Karlsen et al. 2008) using the following search criteria: peptide mass tolerance of 10 ppm, a fragment mass tolerance of 0.6 Da, +2 and +3 charged ions, possible protein post‐translational modifications including oxidation of methionine and propionamidation of cysteine were used during the MASCOT searches. Peptides that could not be assigned to any annotated genes are listed in Table S4.

### Bioinformatical tools

In order to identify potential *c*‐type cytochromes genes in the genome of *M. capsulatus,* an in‐house database containing all putative open reading frames (ORFs) (Karlsen et al. 2008) was searched for the presence of the canonical CxxCH heme‐binding motif (Scott 1996). In total, 206 CxxCH motifs were identified distributed among 70 different ORFs. Bioinformatic analyses suggested that 51 of these ORFs contain a putative leader sequence and/or transmembrane helices indicating a translocation through the gram‐negative inner cell membrane, a prerequisite for the maturation of *c*‐type cytochromes (Thony‐Meyer and Kunzler 1997); Stevens et al. 2011). Further, sequence and homology analyses using PROSITE and BLASTp strongly supported that these 51 ORFs encode *c*‐type cytochromes. The 51 putative *c*‐type cytochromes identified in the genome of *M*. *capsulatus* are summarized in Table S2. Sequence similarity searches were performed in the GeneBank database with BLASTP using default settings (http://blast.ncbi.nlm.nih.gov/Blast.cgi). Protein sequences were scanned for motifs and conserved domains using PROSITE (http://prosite.expasy.org/), SUPERFAMILY (http://supfam.cs.bris.ac.uk/SUPERFAMILY/) and PFAM (http://pfam.xfam.org/), respectively, while SignalP (v3.0) (http://www.cbs.dtu.dk/services/SignalP/), TatP (v1.0) (http://www.cbs.dtu.dk/services/TatP/), SecretomeP (v2.0) (http://www.cbs.dtu.dk/services/SecretomeP/), and TMHMM (v2.0) (http://www.cbs.dtu.dk/services/TMHMM/) were used for prediction of signal peptides, secretion, and transmembrane helixes, respectively. The multiple sequence alignment was generated with CLUSTALX (v2.0) using default alignment parameters, prior to manual editing using the GeneDoc software (http://www.iubio.bio.indiana.edu).

## Results

### Microarray transcriptome profiling

**Figure 1:**
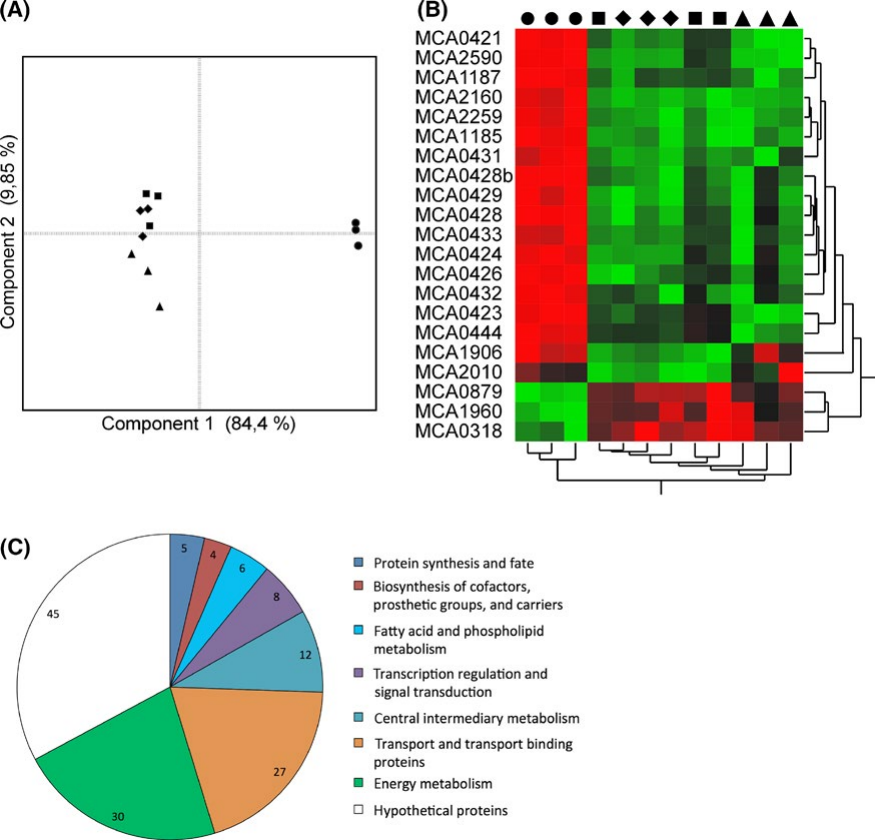
Statistical and functional analyses of the transcriptome responses in *Methylococcus capsulatus* grown under different copper regimes. (A) Principal component analysis of normalized expression values (Log2) of all differentially expressed transcripts. Individual samples are indicated according to the copper concentration during growth (*n* = 3); 0 *μ*mol/L copper (●), 0.8 *μ*mol/L copper (■), 1.6 *μ*mol/L copper (___), 5 *μ*mol/L copper (▲). (B) Two‐ way hierarchal clustering analysis of normalized expression values (Log2) of differentially expressed c‐type cytochromes. *Y*‐axis indicates gene annotation. Individual samples (*X*‐axis) are labeled with symbols as indicated above. (C) Pie chart showing the distribution of functional classifications of 137 differential expressed genes between soluble methane monooxygenase and particulate methane monooxygenase‐producing cells based on Gene Ontology assignments. Multivariate statistical analyses were performed with the JMP 11 software from SAS Institute Inc., (Cary, NC).

Whole‐genome transcriptome profiling with microarrays was used to globally study the changes in gene expression in *M. capsulatus* cells producing either sMMO or pMMO. These analyses were performed on cells grown to the exponential phase (OD_600_ ≈ 0.6) with 0, 0.8, 1.6, or 5.0 *μ*mol/L CuSO_4_ added to the NMS growth medium. Prior to the microarray analyses, cultures were screened for sMMO activity with the naphthalene assay, and as expected, sMMO was only produced in the cultures with no added copper (Brusseau et al. 1990). Principal component analyses of the gene expression data between the different growth regimes revealed that the major changes in expression values were between sMMO‐ and pMMO‐producing cells (Fig. 1). Although the gene expression among the cultures producing pMMO was predominantly uniform, an apparent gradient along the second principal component is observed. This is in accordance with the presence of some genes showing a dose‐dependent response in expression to increasing levels of copper. In total, 87 and 49 genes were significantly up‐regulated and down‐regulated, respectively, in sMMO‐producing cells when compared to pMMO‐producing cells (Table S1). A pie chart showing the distribution of gene ontology groups identified in the microarray analysis as differentially expressed between the sMMO‐ and pMMO‐producing cultures is shown in Figure 1C. As expected, the genes encoding the two versions of MMO showed the highest differential expression, with the alpha subunit of sMMO being 333‐fold up‐regulated, and the B subunit of pMMO sevenfold down‐regulated in sMMO‐producing cells. The most enriched ontology group of differentially expressed genes is related to the energy metabolism, and includes 21 *c*‐type cytochromes. The second largest ontology group contains genes involved in transport functions, which the majority of genes appear to be related to metal ion transport. Of genes involved in the central intermediary metabolism, all but two are directly linked to methane oxidation. Furthermore, six and eight genes are related to the fatty acid metabolism and regulation of transcription, respectively, which most likely are functionally linked to the large morphological difference between pMMO‐ and sMMO‐producing *M. capsulatus* (Prior and Dalton 1985).

**Figure 2:**
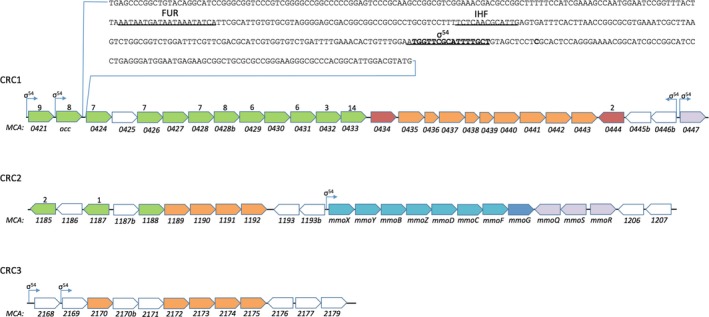
The genetic organization of copper‐responding gene clusters in *Methylococcus capsulatus*. Genomic regions containing clusters of genes and operons with copper responding transcription are depicted. ORFs are colored based on predicted functional categories as green (*c*‐type cytochromes), red (biosynthesis of cofactors), orange (transport), transcription regulation (purple), methane oxidation (blue), chaperones (dark blue), and hypothetical proteins (white). Number of heme‐binding motifs are shown above the individual ORFs, and putative sigma54 promoters are indicated. The sequence upstream of MCA0424 with putative binding sites for FUR, integration host factor (IHF), and RNA polymerase *σ* ^54^ factor is shown in detail.

It was evident from the transcriptome data that there are three copper‐responding gene clusters (CRC) in *M. capsulatus* that strongly respond to copper limitation (Fig. 2). CRC 1 encompasses MCA0421 to MCA0447 and includes several multiheme *c*‐type cytochromes and TonB‐family proteins. TonB systems utilize the proton gradient to actively transport a wide range of substrate over the cell membrane of Gram‐negative bacteria (reviewed in Krewulak and Vogel 2011). CRC 2 encompasses MCA1185 to MCA1207 and includes the soluble methane monooxygenase‐encoding genes, two *c*‐type cytochromes, a pyrophosphatase, and several hypothetical proteins that show sequence similarities to proteins involved in metal storage and transport. CRC 3 encompasses MCA2168 to MCA2179, and encodes several hypothetical proteins, an ABC family transport system with sequence similarity to the high‐affinity zinc uptake system ZnuABC, as well as a *copC* encoding gene. CopC family proteins are located to the periplasm where they have been shown to function as copper chaperones (Djoko et al. 2007).

Importantly, the largest functional group of genes that showed differential expression was the *c*‐type cytochromes where 18 up‐regulated and three down‐regulated transcripts were found in sMMO‐producing cells (Fig. 1B and Table 1). Interestingly, two of the down‐regulated *c*‐type cytochromes in copper‐limited cells are a ubiquinol‐cytochrome *c* reductase (MCA1960) and a cytochrome *c* oxidase (MCA0879), indicating that also major changes occurs in the respiratory chain between sMMO‐ and pMMO‐producing *M. capsulatus*. The possible implications of the observed regulation of these enzymes on the respiratory chain in *M. capsulatus* are discussed in detail later.

**Table 1:** Summary of differential expressed genes encoding *c*‐type cytochromes between soluble methane monooxygenase (sMMO) and particulate methane monooxygenase (pMMO) producing cells of *Methylococcus capsulatus* strain Bath. See also Table S2 for more information

Locus Tag	Annotation/function	Fold change	Detected by MS^a^
MCA0318	MauG family protein	−3.2	ND
MCA0421	Cytochrome *c*_553O_ family protein	13.9	S
MCA0423	Cytochrome *c*_553O_	10.1	S
MCA0424	Multiheme *c*‐type cytochrome	4.8	S
MCA0426	Multiheme *c*‐type cytochrome	8.6	S
MCA0428	Cytochrome *c*_3_ family protein	7.2	ND
MCA0428b	Cytochrome *c*_3_ family protein	3.7	ND
MCA0429	Multiheme *c*‐type cytochrome	5.4	ND
MCA0431	Multiheme *c*‐type cytochrome	4.3	ND
MCA0432	Multiheme *c*‐type cytochrome	2.7	ND
MCA0433	Multiheme *c*‐type cytochrome	2.3	ND
MCA0444	MauG family protein	2.7	S, OM^b^
MCA0879	Subunit II of terminal cytochrome *c* oxidase	−1.8	IM^c^
MCA1185	Diheme cytochrome *c*_4_ family protein	13.8	IM, OM^b^
MCA1187	Membrane‐embedded *c*‐type cytochrome	28.3	IM, OM^b^
MCA1906	MauG family protein	14.7	S
MCA1960	Cytochrome *c*_1_ subunit of ubiquinol‐cytochrome *c* reductase	−2.6	ND
MCA2010	Predicted *c*‐type cytochrome	3.2	ND
MCA2160	Cytochrome *c*_553O_ family protein	9.0	ND
MCA2259	Cytochrome *c*_553O_ family protein	11.3	S
MCA2590	MauG family protein	12.0	S

a Peptides were analyzed with LTQ‐ORBITRAP. S, surface fraction; P, periplasma fraction; IM, inner membrane fraction; OM, washed outer membrane fraction; ND, not detected.

b Only detected in sMMO‐producing cells.

c Only detected in pMMO‐producing cells.

**Figure 3:**
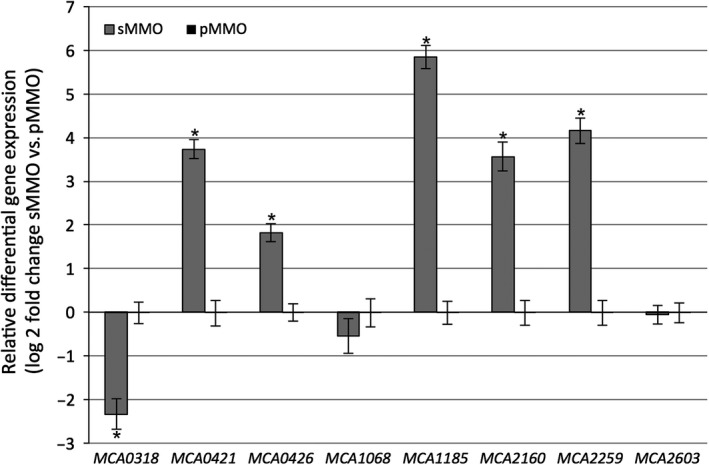
Quantitative RT‐PCR validation of differential relative gene expression levels of selected *c*‐type cytochromes in sMMO‐producing cells. RNA levels are normalized to *mopB* and shown as expression values relative to cells grown with 0.8 *μ*mol/L CuSO_4_. The mean ± SD is shown for three independent biological and two technical replicates. *P < 0.01 using a Student's *t*‐test.

qRT‐PCR was subsequently used to analyze a selected set of the *c*‐type cytochromes to confirm the data obtained from the global transcript analysis. The results are summarized in Fig. 3. For clarification, only the difference in expression between the cultures with 0 and 0.8 *μ*mol/L copper added to the growth medium is shown, as no significant differences in gene expression was observed among the pMMO‐producing cultures. In accordance with the microarray data, these analyses confirmed that the transcription of MCA0318 decreased, and that the transcription of MCA0421, MCA0426, MCA1185, and MCA2160, and MCA2259 increased in sMMO‐producing cells. Of the dihaem *c*‐type cytochromes that are likely involved in electron transport, MCA1185 showed a copper‐dependent expression, while MCA1068 and MCA2603 were constitutively expressed, in accordance with the microarray data.

### The c‐type heme proteome in *M. capsulatus*

**Figure 4:**
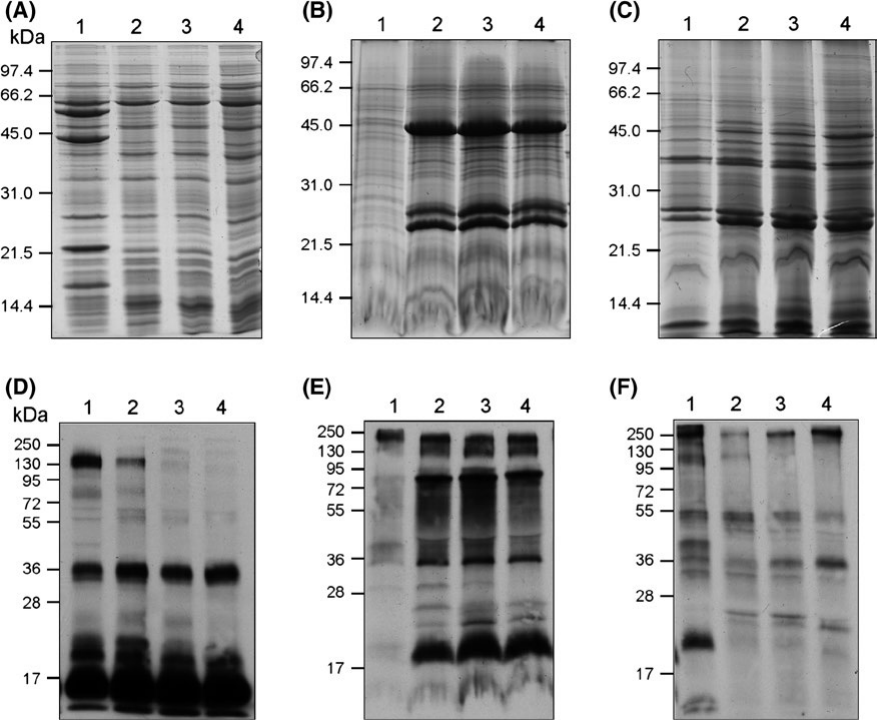
Nonreducing SDS‐PAGE analyses of the protein fractions obtained from cultures grown at different copper concentrations. The *Methylococcus capsulatus* cultures were grown with copper concentrations corresponding to ~0 (lane 1), 0.8 (lane 2), 1.6 (lane 3), and 5 *μ*mol/L (lane 4) copper. A 12.5% polyacrylamide gel were used and stained with CBB of enriched protein fraction of periplasm (A), inner membrane (B), and outer membrane (C). (D, E, and F) *c*‐type heme staining of (A, B, and C, respectively) using enhanced chemiluminescence (ECL, GE) on nitrocellulose membranes.

To further substantiate the copper‐dependent expression of the *M. capsulatus c*‐type cytochromes identified in the transcriptome data, different proteomic analyses were conducted on cells from the same cultures as used in the microarray analyses. Enriched protein fractions, including the cell surface proteins, the periplasm, and the inner‐ and outer membranes, were obtained as previously described (Karlsen et al. 2005, 2008). The *c*‐type cytochromes in the enriched cellular fractions were subsequently assessed by *c*‐type heme staining of SDS‐PAGE‐separated proteins transferred onto nitrocellulose membranes (Fig. 4). In line with the microarray data, the most significant differences in the protein pattern were found between sMMO‐ and pMMO‐producing cells, clearly emphasizing the major effect the presence of copper has on the *M. capsulatus* proteome. The surface‐associated *c*‐type cytochromes of *M*. *capsulatus* were extensively characterized by Karlsen et al. (2008), and a similar expression profile was observed in the present work (data not shown). From Fig. 4D, we show that the enriched periplasmic fractions is dominated by low‐molecular weight *c*‐type cytochromes in the range of 6–36 kDa, and that the abundance of these cytochromes appears not to vary significantly between cells grown with 0 to 5 *μ*mol/L CuSO_4_ in the growth medium. However, the expression of less abundant *c*‐type cytochromes appeared to respond to different concentrations of copper in the growth medium, and several, in particular those of ~140, ~80, and ~20 kDa, increased in abundance in sMMO‐producing cells. This observation is similar to the expression pattern observed for the surface‐located *c*‐type cytochromes (Karlsen et al. 2008). Interestingly, an opposite trend was observed in the inner membrane fractions (Fig. 4E). Besides the high‐molecular‐weight *c*‐type cytochromes, sMMO‐expressing cells are almost depleted of *c*‐type cytochromes in this cellular compartment when compared to pMMO‐expressing cells. Moreover, in pMMO‐expressing cells, *c*‐type cytochromes are abundant in the inner membrane fraction at ~80, ~35, and ~20 kDa and they appear not to vary significantly in expression with increasing copper concentrations. However, less abundant *c*‐type cytochromes in the inner membrane fraction show a copper‐dependent expression, which increases (at ~22 kDa) and decreases (at ~28 kDa) with increasing copper concentrations. The total amount of *c*‐type cytochromes appear to be less abundant in the outer membrane fraction than in the other fractions, and also in this fraction the major differences in the pattern of *c*‐type cytochromes are between sMMO‐ and pMMO‐expressing cells (Fig. 4F). In the outer membrane fraction of sMMO‐expressing cells, there are *c*‐type cytochromes at ~300, ~40, and ~20 kDa that apparently are not present in pMMO‐expressing cells, while there are no observed differences in abundance of *c*‐type cytochromes among the protein profile of the different pMMO‐expressing cultures.

**Figure 5:**
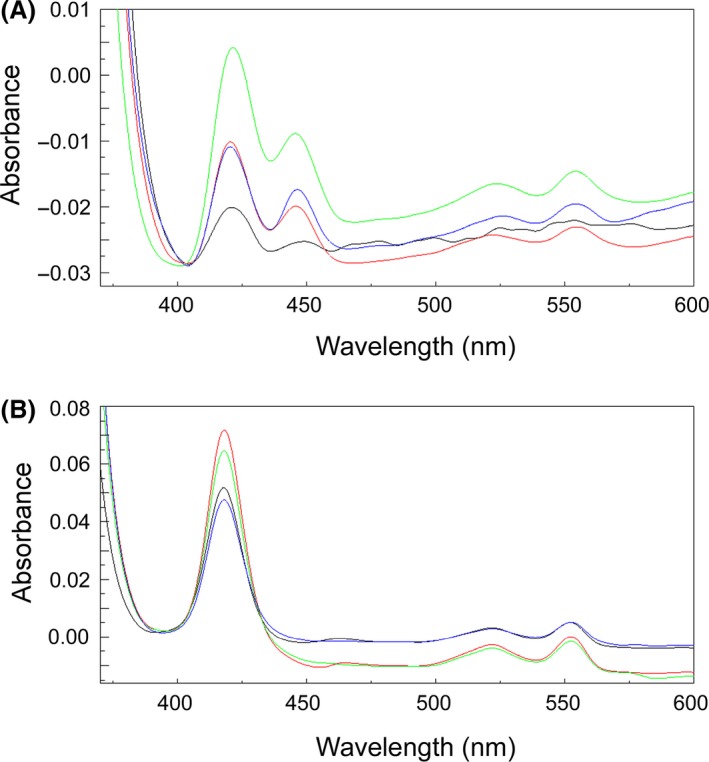
Reduced‐minus‐oxidized difference spectra of the inner membrane (A) and periplasmic (B) fractions. Spectra were obtained from the ~0 (black), 0.8 (red), 1.6 (green), and 5 (blue) *μ*mol/L copper cultures.

In order to estimate the content of *c*‐ type cytochromes in the cellular fractions, reduced‐minus‐oxidized spectra of the periplasmic‐ and inner membrane fractions were recorded between 300 and 600 nm (Fig. 5). Due to the particulate nature of the outer membrane fraction, redox spectra could not be recorded for these samples. The resulting spectra revealed local maxima at 420, 520, and 550 nm in the cellular fractions analyzed, which are characteristic signatures for *c*‐type heme proteins (Ingledew 1982; Gennis 1987). Furthermore, the redox spectra support the findings obtained by the heme staining, that is, when copper is present, its concentration in the growth medium affect to a lesser extent the amount of *c*‐type cytochromes in the periplasm, and that there is a significant difference in the amount of *c*‐type cytochromes present in the inner membranes of sMMO‐ compared to pMMO‐producing cells. Estimates based on the differences between absorbance values at the Soret‐peak (403 and 421 nm) (Niederman 1974) indicate that the amount of *c*‐type cytochromes in the inner membrane fraction is approximately 3.5‐fold lower in sMMO‐producing cells compared to cells grown with 5 *μ*mol/L copper added to the growth medium (Fig. 5). This is in contrast to the fivefold enrichment of surface‐located *c*‐type cytochromes observed in sMMO‐expressing cells (Karlsen et al. 2008), and demonstrate that copper starvation affects the *c*‐type cytochromes located to the inner membranes differently than those associated to the cell surface of *M. capsulatus*. Interestingly, the local maximum at 440 nm in the inner membrane fractions, typical of *a*‐type heme (Vanneste 1966), becomes prominent in pMMO‐producing cells, and increases with increasing amount of copper in the growth medium (Fig. 5). This increase is probably due to an increased expression of cytochrome *caa*_3_, a well‐known copper‐containing enzyme (Ferguson‐Miller and Babcock 1996), and a likely source of *a*‐type heme as well as the major source of *c*‐type cytochrome in the inner membrane of *M*. *capsulatus* (DiSpirito et al. 1994). Importantly, it is clear that copper affects the expression of several different *c*‐type cytochromes in *M*. *capsulatus* and that this effect is not limited to the surface‐located *c*‐type cytochromes reported previously (Karlsen et al. 2008).

### Identification of *c*‐type cytochromes with LTQ‐ORBITRAP mass spectrometry

In order to identify expressed *c*‐type cytochromes and their subcellular localization in *M. capsulatus*, LTQ‐ORBITRAP mass spectrometry was used to analyze the protein content of the enriched fractions shown in Fig. 4, including surface‐extracted proteins obtained as described previously (Karlsen et al. 2008). In total, we identified peptides that could be assigned to 24 different *c*‐type cytochromes. The results are summarized in Table S2.

In the surface‐extracted protein fraction, we identified three of the five cytochrome *c*_553O_ paralogues and the diheme MCA2590 previously identified from the surface of *M*. *capsulatus* (Karlsen et al. 2005, 2008). In addition, we identified MCA0424, MCA0426, and MCA1906. MCA0424 and MCA0426 are located in CRC 1 and possibly form an operon of eleven genes, including eight *c*‐type cytochromes with a potential of containing a total of 58 heme groups. BLASTp homology searches showed that MCA1906 shares sequence similarity to the BCCP/MauG‐family of *c*‐type cytochromes (Fig. S1). MauG‐like *c*‐type cytochromes are so far found to be involved in the biosynthesis of prosthetic groups (Wang et al. 2003). The highest diversity of *c*‐type cytochromes were observed in the periplasmic fractions where nine different *c*‐type cytochromes were identified, including cytochrome *c*_555_, cytochrome P460, cytochrome *c* peroxidase (Ambler et al. 1986; Zahn et al. 1994, 1997), as well as MxaG, the electron acceptor for methanol dehydrogenase. In addition, two diheme (MCA1068 and MCA2603) and five monoheme (MCA0940, MCA1585, MCA1775, MCA2181, and MCA2405) *c*‐type cytochromes were identified. MCA1068, MCA2603, and MCA0940 show sequence similarities to cytochrome *c*_554_ from *Vibrio parahaemolyticus* and cytochrome *c*_4_ from *Pseudomonas stutzeri* (Kadziola and Larsen 1997; Akazaki et al. 2010). MCA0940 differs from other class I cytochromes *c* by having an extended region between the CxxCH motif and the methionine forming the sixth heme‐coordinating ligand. Interestingly, MCA2181 appears to be a fusion protein between a class I cytochrome *c* and a conserved hypothetical protein. MCA1585 is a fatty acid *cis‐trans* isomerase previously characterized from *M. capsulatus* (Löffler et al. 2010). Of the *c*‐type cytochromes previously characterized, only cytochrome *c*_553_ has not been sequence identified (Ambler et al. 1986; Zahn et al. 1994). Based on the reported molecular mass of ~10.7 kDa and pI of 8.8, this *c*‐type cytochrome is most likely encoded by MCA2405 (theoretical mass of 9.4 kDa and pI of 9.0). MCA2405 show high sequence similarity to cytochrome *c*_552_ from *Nitrosomonas europaea*, a small *c*‐type cytochrome with a His/Met coordinated heme able to function in a wide range of electron transfer reactions (Timkovich et al. 1998).

Considerable fewer *c*‐type cytochromes were identified in the inner and outer membrane enriched fractions. However, the observed increase in *a*‐type cytochromes in the redox spectra of the inner membranes with increasing copper concentrations in the growth medium, were also reflected in our mass spectrometry results where peptides belonging to subunit II of cytochrome *caa*_3_ were only detected in pMMO‐producing cells. Interestingly, in copper‐limited cells, two new *c*‐type cytochromes, MCA1185 and MCA1187, appear in the inner membrane fraction. MCA1185 is a diheme cytochrome paralogous to MCA1068, while MCA1187 is an integral membrane protein with eight predicted transmembrane helices and with a C‐terminal *c*‐type cytochrome domain. MCA1187 is thus the putative source of the residual *c*‐type heme signal seen in Fig. 4. MCA1185 and MCA1187 are located in CRC 2 and may form an integral membrane complex with MCA1186. Sequence analyses shows that MCA1187 is related to the cytochrome *bd*‐family of copper‐independent terminal quinol oxidases (Fig. S2).

## Discussion

Transcriptomic profiling was used to investigate the gene expression pattern in *M. capsulatus* during methanotrophic growth in the presence and absence of copper. In total, 137 ORFs were found to be differentially expressed between cells producing sMMO and pMMO, while only minor differences in gene expression were observed between the pMMO‐producing cultures. Of these, 87 genes were found to be up‐regulated during sMMO‐producing cells, that is, during copper‐limited growth. This is a considerable higher number of genes compared to that previously found for *Pseudomonas aeruginosa* and *Bradyrhizobium japonicum* where only ten and five genes, respectively, showed increased expression during copper limitation (Frangipani et al. 2008; Serventi et al. 2012). However, it is well known that methanotrophs possessing sMMO are highly adapted to copper starvation. It has been known for some time that the “copper switch” is not only limited to the expression of the MMO's, but also affect a range of different proteins (Kao et al. 2004). From the microarray analyses performed in this study, the two largest functional groups of genes that varied their expression in pMMO versus sMMO producing cells were related to the energy‐ and transport metabolisms. This was also found as the two most enriched functional ontologies in a quantitative proteomic analysis of *M. capsulatus* grown in a fermentor with either 0 or 30 *μ*mol/L copper added to the growth medium (Kao et al. 2004; Karlsen et al. 2003, 2005, 2008). Besides 21 *c*‐type cytochromes showing differential expression between sMMO‐ and pMMO‐producing cells, we also observed changes in transcript levels for several genes that are functionally related to the respiratory chain. Moreover, there was reduced expression of the cytochrome *caa*_3_, which also was evident from the diminishing signal of heme *a* at 440 nm in the reduced‐oxidized difference spectrum of the inner membrane fraction of sMMO‐producing cells. Cytochrome *caa*_3_ is a copper‐containing enzyme and a similar response to copper limitation was previously observed in *P. aeruginosa* (Frangipani et al. 2008). Moreover, in *P*. *aeruginosa,* the terminal oxidase *cioAB* was found to be up‐regulated in copper‐starved cells. CioAB belongs to the cytochrome *bd*‐family of copper‐independent terminal quinol oxidases (Cunningham et al. 1997). The microarray analyses showed that the MCA1187‐encoding gene dramatically increased its expression in cells producing sMMO. Furthermore, MCA1187 was only detected by LTQ‐ORBITRAP MS in the inner membrane fraction obtained from sMMO‐producing cells (Table 1). A multiple alignment of MCA1187, MCA1187‐like proteins, and CioA homologs revealed interesting similarities and differences between these two groups of proteins (Fig. S2). The prediction and localization of the transmembrane helixes 1 to 8, histidine 19; (CydA *E*. *coli* numbering; Borisov et al. 2011) coordinating heme *b*_595_, and glutamate 99 and 108; coordinating heme *d* in helix 4 are positionally conserved among all amino acid sequences. However, the quinone loop that are present in CioA, histidine 186, and methionine 393 coordinating heme *b*_558_, as well as transmembrane helix 9 are not conserved in MCA1187, or in the other MCA1187‐related sequences obtained from other species. Furthermore, a CxxCH motif is located in the C‐terminal part of MCA1187 and MCA1187‐like proteins, but not in the CioA homologs. Taken together, this indicates that quinone is not the electron donor for MCA1187 and MCA1187‐like proteins, and that a cytochrome *c* domain possible has replaced heme *b*_558_. Amino acid residues involved in coordinating heme *b*_595_ and heme *d* that forms the active site in cytochrome *bd* are still conserved in MCA1187‐like proteins. We therefore postulate that MCA1187‐like proteins may function as copper‐independent terminal cytochrome *c* oxidases forming a novel family within the cytochrome *bd*‐family as cytochrome *cbd* proteins.

**Figure 6:**
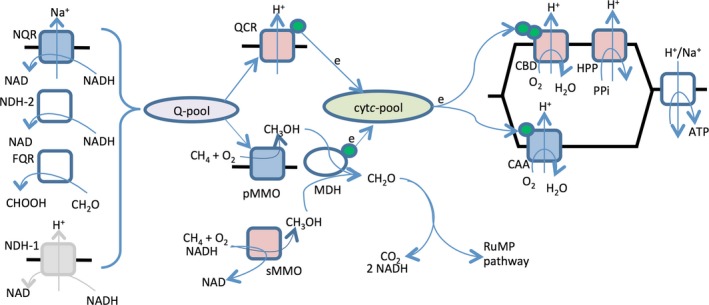
Proposed pathway of methane oxidation in *Methylococcus capsulatus* cells producing either sMMO or pMMO. Proteins showing positive or negative Cu regulation are shown in blue and red, respectively. Proteins not showing a Cu regulation or have low expression values (based on microarray data) are shown in white and gray, respectively. *c*‐type cytochromes are shown in green. NQR, Na^+^‐translocating NADH‐quinone reductase; NDH‐2, type 2 NADH dehydrogenase; FQR, formaldehyde‐quinone reductase; NDH‐1, type 1 NADH dehydrogenase; QCR, quinol‐cytochrome *c* reductase; PMMO, particulate methane monooxygenase; SMMO, soluble methane monooxygenase; MDH, methanol dehydrogenase; CAA, cytochrome *caa*_3_; CBD, cytochrome *cbd*; HPP, H^+^‐translocating pyrophosphatase; ATP, ATPase.

Other genes linked to the *M. capsulatus* respiratory chain showing differential expression between sMMO‐ and pMMO‐producing cells, were the Na(+)‐translocating NADH‐quinone reductase and the ubiquinol‐cytochrome *c* reductase genes. We did not observe a copper‐dependent expression of the membrane‐associated quinoprotein formaldehyde dehydrogenase and the type 2 NADH dehydrogenase, previously suggested as being initial electron donors to pMMO (Zahn et al. 2001; Cook and Shiemke 2002). Partly purified pMMO is shown to utilize quinone as electron donor, either directly or via a cytochrome intermediate (Zahn and DiSpirito 1996). Based on our data of differentially expressed genes, we hypothesize a model for how energy transduction occurs in pMMO‐ and sMMO‐producing cells (Fig. 6). In line with previous experimental data, we suggest that quinone is predominantly used by pMMO in order to sustain its activity. This would also imply that electrons for the oxygen reductase are mainly delivered from methanol dehydrogenase. However, considering the many *c*‐type cytochromes expressed in *M. capsulatus,* we can currently not exclude the possibility that also cytochromes can provide electrons to pMMO. During sMMO production, we suggest that a more common electron transport chain is present, where electrons from quinone are channeled via ubiquinol‐cytochrome *c* reductase to oxygen reductase with possibly MCA1187 as the primary terminal oxygen reductase. Since one of the NADH molecules generated from oxidation of formaldehyde is used by sMMO, the pyrophosphatase located in CRC 2 might be involved in maintaining an electrochemical gradient across the cell membrane. This can also be one of the reasons for the lower growth yield observed for *M. capsulatus* when expressing sMMO (Leak and Dalton 1986).

Two high‐affinity transport systems for copper have been described in methanotrophs; the secreted chalcophore methanobactin, and the homologous CorA and MopE proteins isolated from *M. album* BG8 and *M. capsulatus,* respectively (Berson and Lidstrom 1997; Karlsen et al. 2003; Kim et al. 2004). Currently, there are conflicting evidences to whether *M. capsulatus* is able to synthesize methanobactin (Choi et al. 2010; Kenney and Rosenzweig 2013). It has been shown that MopE binds copper (Ve et al. 2012), but nothing is known about how copper is transferred from MopE to the cell interior. Our microarray data revealed two putative transport systems that have an increased expression during copper limitation. This includes a TonB system located in CRC 1 and an ABC transporter system located in CRC 3. Both these gene systems show low sequence similarities to characterized transporters in other bacteria that are available in gene databases, but include both iron‐ and copper‐specific receptors. The presence of a CopC homolog in CRC 3 may also suggest that the ABC transporter located in CRC 3 is involved in copper acquisition. Furthermore, based on genomic context, it has been suggested that methanobactin is taken up by the cells through a TonB system in methanotrophs (Kenney and Rosenzweig 2013). The TonB receptor located in CRC 1 (MCA0440) has only limited sequence identity to the suggested methanobactin receptor in *Methylosinus trichosporium*, but at present, we cannot exclude that MCA0440 may be involved in copper acquisition.

Intriguingly, the majority of the *c*‐type cytochromes that increased their expression in sMMO‐producing cells were identified by LTQ‐ORBITRAP MS at the cell surface. This includes the cytochrome *c*_553O_ family of multiheme *c*‐type cytochromes and the diheme SACCP which previously have been reported to be noncovalently attached to the *M*. *capsulatus* cell surface (Karlsen et al. 2005, 2008). In this study, we also identified MCA0424 and MCA0426 in the surface‐enriched fraction isolated from sMMO‐producing cells. MCA0424 and MCA0426 are part of a putative operon comprising of eleven genes of which eight are multiheme *c*‐type cytochromes, containing a total of 58 CxxCH motifs. Upstream of MCA0424, a promoter region containing recognizable sigma70‐ and sigma54 (ATGGTTCGCATTTTGCT) promoter‐like sequences, a putative integration host factor sequence (TCTCAACGCATTG), and a putative FUR box (AATAATGATAATAAATATCA), can be predicted and indicate a complex regulation of transcription of this operon (Hawley and McClure 1983; Collado‐Vides et al. 1991; Barrios et al. 1999; Baichoo and Helmann 2002). FUR is a metal‐dependent regulator of transcription that senses metal concentration and/or redox state of the cells (reviewed in Lee and Helmann 2007). The presence of a *beta*‐barrel outer membrane protein encoded by MCA0427 in this operon is also interesting. Surface‐located multiheme *c*‐type cytochromes and beta‐barrel outer membrane proteins are intimately linked to the ability to utilize extracellular electron acceptors (Richardson et al. 2012). In *Shewanella*, the beta‐barrel protein MtrB forms a porin in the outer membrane allowing close contact between the *c*‐type cytochromes MtrA and MtrC located to periplasm and cell surface, respectively (Ross et al. 2007; Clarke et al. 2011). Reconstitution of MtrABC in liposomes has also demonstrated that this complex is sufficient for conducting electrons over the membrane (Hartshorne et al. 2009). At present, no biochemical evidence for any biological function for this MCA0424‐system in *M*. *capsulatus* exist, but it is highly plausible that this system would allow the transfer of electrons to the exterior. Obviously, further analyses are necessary to elucidate the physiological function of this intriguing gene cluster.

The high number of *c*‐type cytochromes in *M. capsulatus* is puzzling, but not necessarily surprising. Genome comparisons have shown that the proportion of gene duplications and the presence of paralogous genes are increased in species with a highly specialized metabolism, like *M. capsulatus*, compared to species that are able to utilize a wide variety of substrates for growth (Mahadevan and Lovley 2008). We have in this study shown that the majority of the *c*‐type cytochromes are expressed in *M. capsulatus*, indicating that they are an important and integral part of its metabolism.

## Conclusion

The high number of up‐regulated genes in sMMO‐producing cells shows that *M*. *capsulatus* is highly adapted to copper‐limited growth. Besides the switch in expression of the MMOs, our data revealed that the “copper switch” also includes major changes to the respiratory chain. Our data strongly suggest data during pMMO production, the majority of quinones are directed to methane oxidation in line with what has been previous observed previously. Furthermore, in total, 35 *c‐*type cytochromes were detected at the protein level, indicating that these proteins are an integral part of the *M. capsulatus* metabolism. Interestingly, the transcription of numerous *c*‐type cytochrome‐encoding genes responded to copper limitation. Eight of the regulated *c*‐type cytochromes were located to the cell surface, indicating putative roles in extracellular electron transfer reactions. Although, one should be cautious in interpreting gene function solely from gene sequence, we think that the combination of bioinformatic analyses, subcellular localization analyses, and the expression studies presented here, have provided data that warrant further studies on the specific functions of the *c*‐type cytochromes of *M. capsulatus*.

## Conflict of Interest

None declared.

## Supporting information


**Figure S1.** Condensed multiple sequence alignment for known MauG proteins and bacterial di‐heme cytochrome *c* peroxidase proteins to the homologs in *M. capsulatus*.

**Figure S2.** Alignment of MCA1187 to cytochrome *bd* subunit I and homologs.

**Table S1.** List of differentially expressed genes between cells producing sMMO and pMMO.
**Table S2.** Summary of genes encoding *c*‐type cytochromes in *M*. *capsulatus*.
**Table S3.** List of primers used in qRT‐PCR.
**Table S4.** List of unassigned peptides from the LTQ‐ORBITRAP mass spectrometry analyses of the *M. capsulatus* cellular ffractions.
