# Supplementary material for: Transcriptomic profiling of Methylococcus capsulatus (Bath) during growth with two different methane monooxygenases
Source: Microbiologyopen. 2015 Dec 20;5(2):254–67. doi: 10.1002/mbo3.324 (PMC4831470; doi:10.1002/mbo3.324)
Supplement: Supplementary file 1 — Figure S1. Condensed multiple sequence alignment for known MauG proteins and bacterial di‐heme cytochrome c peroxidase proteins to the homologs in M. capsulatus. [file MBO3-5-254-s001.docx]

* 20 * 40 * 60
MauG_Pn : .[32]LGAQLFVDPALSRNATQSCATCHDPA....RAFTDPREGKAGLAVSV......GD : 77
MauG_Me : .[69]LGKALFFDPRLSRSGSVSCATCHNPS....LGWSDGLTRAVGFGMVP........
MauG_Mf : .[50]LGKTLFFDPRLSRDGSMSCATCHNPG....MRWSDGRILPLRADGVE........
MauG_Mm : .[57]LGKTLFFDARLSRDNSMSCATCHAPD....KRWSDGRLRPLGSEHVA........
MauG_Mt : .[35]LGKALFFDPRLSREKGMSCATCHSPD....HRWSDGRVVPLGSEELE........
AAR34912 : .[30]LGKDIFFDTNLSINGNQSCADCHAPE....AGWTGPTSEVNA.HGAVY....EGS
MCA1769 : .[35]LGKALFFDPSLSTPPGQSCADCHDPK....AGWTGSDQDINL.HGGVY....EGA
MCA1906 : .[38]LGQKLFFDTNLSTPPGQACSSCHDPA....TAFSDPNKSQPTSNGA.........
EGL02226 : .[38]LGQQLFFDTHLSEPPGQACATCHNPA....TAFTDADKSRPTSKGV.........
AEG01769 : .[27]LGERLFSDKNLSLNKNQACASCHSLS....PAHAKFPQTKRVPGFVDA[10]NGS
CcpA_Pa : .[55]LGKKLFFDPRLSRSHVLSCNTCHNVG....TGGADNVPTSVG.HGWQK.......
Ccp_Pp : .[46]LGKVLFFDPRMSSSGLISCQTCHNVG....LGGVDGLPTSIG.HGWQK.......
CcpA_Rc : .[56]LGAMLFFDPRMSKSGVFSCQSCHNVG....LGGVDGLETSIG.HGWQK.......
Ccp_Mh : .[55]LGKMLFFEPRLSSSHLISCNTCHNVG....LGGDDELPTSIG.HGWQK.......
Ccp_Ne : .[46]LGKMLFFDPRLSKSGFISCNSCHNLS....MGGTDNITTSIG.HKWQQ.......
CcpA_Gs : .[54]LGKMLYFDPRLSASHLISCNTCHNVG....LGGGDLQATSTG.HGWQK.......
MCA0345 : .[44]LGKMLYLDPRLSSTGTVSCNSCHNVM....LGGEDNRGGSVGVHG.QV.......
AEG01939 : .[43]LGKMLYHDPRLSSTGTVSCASCHNTM....LGGEDNRPNSMGVNG.QT.......
EGL01965 : .[44]LGKMLYHDPRLSSTGTVACASCHNTM....LGGEDNRPNSMGVNG.QT.......
EFO07874 : .[44]LGQMLYHDPRLSSTGTVSCSSCHNTM....LGGEDNRPNSMGVNG.QT.......
EEF80583 : [336]LGKKLFFDRRLSLNNTISCAICHVPE....QGFTNHEILTAVGFEGRS.......
EEF81059 : [335]LGKKLFFDRRLSLNNTISCAICHVPE....QGFTNHEILTAVGFEGRS.......
MCA0318 : [296]LGRKLFYDRRLSLNQTFSCAMCHIPE....QGFTSQEQATATGIEGRT.......
AEG02508 : [296]LGRKLFYDRRLSFNNTFSCAICHIPE....QGFSSNEMATAVGIEGRS.......
EGL04739 : [304]LGRKLFYDRRLSLNKTFSCAMCHIPE....QGFTSNEMATAVGVEGRT.......
MCA0444 : [239]RGRQIFVEETFDGNG.RTCATCHRPD....NNHTIDPRYIAKLPPSDP.[3]AEY
AEG01086 : [248]KGRHIFHNETFAGNG.RTCGTCHRAD....NNFTLDPNYIMSLPPTDP.[3]AET
ABA58262 : .[54]QGRDIFFNETFGGNG.RTCSTCHRAE....ANFTIDPEFISALPSNDP.[3]AEF
EGL03975 : .[66]LGKALFWDGNVGSSG.TACASCHFHA[77]FVAVDATGINAGINNDQC[10]GSL
MCA2590 : .[74]LGKALFWDMNVGSDG.MACASCHFHA[69]TAETDNVMSSSGTYAGEF[24]GGI
AEG01090 : .[56]LGKALFWDVNVGSDG.MACASCHFHA[57]IFETDDAVASSGTFSGEF[23]NGI
ABA76906 : .[75]LGKALFWDMEIGSDGSTACASCHYHA[75]VYSTDDVVGSQGVFDANF[21]GGI
 * **

 * 80 * 100 * 120
MauG_Pn : DGQSHGDRNTPTLGYAALVPAFHRDA..NGKYKGGQFWDGRADD....LKQQAGQPMLNP : 131
MauG_Me : .....LPRRTPPVLNLA..............WGTAFQWDGRADS....LEAQARMPITAP
MauG_Mf : .....HARRTPTVLNSA..............WLTTLMWDGRATS....LEDQAILPITTA
MauG_Mm : .....NARRTPTVLNSA..............WLSALMWDGRAGS....LEAQAVLPITTP
MauG_Mt : .....QPRRSPTVMNSA..............WLKALMWDGRANS....LEAQAVLPITTP
AAR34912 : IAGRFGNRKPPSSAYATTAPILKYIRQGGGMFVGGNFWDGRATG.[6]AADQAQGPFLNP
MCA1769 : VATRFGNRKPPTAAYASFSPKFHRDG..NGEFVGGNFWDGRATG.[6]AADQAQGPFLNP
MCA1906 : LKTLKGNRNAPTAAYAAYTPPFHYDP.ILRLFVGGQFLDGRAPT....LKEQAKGPFLNP
EGL02226 : IAGLLGNRNTPTAMYSAYAPAFHFDR.GEGLYFGGQFLDGRAST....LADQAKAPFLNP
AEG01769 : IAGLTGNLNAPSAGYAAHSPEFHWDD.EEGLYIGGQFWNGRAAD....LTEQAKMPFLNP
CcpA_Pa : .....GPRNSPTVFNAV..............FNAAQFWDGRAKD....LGEQAKGPIQNS
Ccp_Pp : .....GPRNAPTMLNAI..............FNAAQFWDGRAAD....LAEQAKGPVQAG
CcpA_Rc : .....GPRNAPTALNAV..............FNVAQFWDGRAPD....LAAQAKGPVQAG
Ccp_Mh : .....GPRNSPTVFNAV..............FNAAQFWDGRAAD....LAEQAKGPVQAG
Ccp_Ne : .....GPINAPTVLNSS..............MNLAQFWDGRAKD....LKEQAAGPIANP
CcpA_Gs : .....GPRNAPTVLNSV..............FNTAQFWDGRAKD....LAEQAKGPVQAP
MCA0345 : .....GGRSAPTVWNAA..............FNSVQFWDGRAPS....LEAQAKGPVTNP
AEG01939 : .....GGRSAPTVWNAA..............FNKVQFWDGRADS....LEAQAAGPVTNP
EGL01965 : .....GGRSAPTVWNAA..............FNKVQFWDGRAES....LEAQAAGPVTNP
EFO07874 : .....GGRSAPTVWNAA..............FNKVQFWDGRAAS....LEAQAAGPVTNP
EEF80583 : .....VKRNAPTIYNTA..............YFKKLFHDGRETS....LEHQAWQPMLAR
EEF81059 : .....VKRNAPTIYNTA..............YFKKLFHDGRETS....LEHQVWQPMLAR
MCA0318 : .....VRRNAPTLYNVA..............YLTKLFHDGRESS....LENQVWGPFLAA
AEG02508 : .....VRRNSPSLYNVG..............YAQLLFHDGRENS....LEQQVWGPLLAH
EGL04739 : .....VRRNSPTLYNVA..............YLDQLFHDGRETA....LERQAWGPLLAH
MCA0444 : NPDL.KELEKPALLRQMG.............LILA.NVDGFDKP.[9]LALPTSIDVEVC
AEG01086 : NPAL.AELENPVLMRKHG.............LILT.NVDGPNVD.[5]PHTLALSTTIAK
ABA58262 : NPSLSKNFEKPEFMHSSG.............LILE.NLDGFQDL.[3]FVLRGSLPTLAL
EGL03975 : NTRQVTKRNAPTVINAAFN............F..RNFWDGRANN[37]LASQAVGPPLDM
MCA2590 : RTRQVEPRNTPTVINAA..............LFHRQFWDGRANS[36]LASQAVGPPVNN
AEG01090 : GTRRVEPRNAPSVINAVFN............H..RSFWDGRGNN[37]LASQAVATAMSQ
ABA76906 : NVRRSTGRNAPSVINAAFN............V..RNFWDGRANN[38]AASQAVGPPGSP
 * *

 * 140 * 160 * 180
MauG_Pn : VEMAMPDRAAVAAR....LRDDPAYRTGFE.ALFGK....GVLDDPERAFDAAAEALAAY : 183
MauG_Me : DEMNMSMD.LVVER....LKAVPGYAPLFR.NAFGS........EEPIGARHVTAALATF
MauG_Mf : HEMNF.EMPLLLNR....LKDVAGYAPLFA.RAFGD.........AEITEKRLTQALASF
MauG_Mm : HEMNF.DMPSVVAR....LESIQGYRPLFT.QAFGD.........ATVNKKRITEALASF
MauG_Mt : HEMNY.NMKELVQR....LGEIKGYQPLFE.DAYGD.........ADITTQRIAMALATF
AAR34912 : LEQGLPDSACVVHR....VCT.ATYGTAME.TLWPG[25]PNRAASDLAYDYIALAIAAY
MCA1769 : LEQNDPSAADVCRK....VAA.SGFAAQLTGSSYPD[10]LDCDNSSDTYDRIALAIAAY
MCA1906 : LEMANPDKQTVVNK....VRE.AEYAWMFD.EVFGP....GSLDNTRKAYDRIAAAIAAF
EGL02226 : IEMANPDPGTVVDK....VRN.AAYAAMFD.TVYGA....GALNDNGVAYDRIADAIAAF
AEG01769 : VEMAMPSELAVVNR....LKQDKTYRRLFR.EVYGL.[9]DQAANAARIFQAAAQAISAY
CcpA_Pa : VEMHS.TPQLVEQT....LGSIPEYVDAFR.KAFPK.......AGKPVSFDNMALAIEAY
Ccp_Pp : VEMSN.TPDQVVKT....INSMPEYVEAFK.AAFPE.......EADPVTFDNFAAAIEQF
CcpA_Rc : VEMNN.TPENLVAT....VQSMPGYVEAFA.KAFPG.......QKDPISFDNFALAVEAF
Ccp_Mh : VEMSS.TPDRVVAT....LKSMPEYIERFE.DAFPG.......QENPVTFDNMAVAIEAY
Ccp_Ne : KEMAS.THEIAEKV....VASMPQYRERFK.KVFGS.........DEVTIDRITTAIAQF
CcpA_Gs : VEMNN.TPDQVVKT....LNSIPDYVALFK.KAFPG.......EKDPVTFDNMAKAIEVF
MCA0345 : IEMGMKSWDDVVAR....LKAIPGYPEAFA.AAFGS........GDAVTADNAAKAIAAY
AEG01939 : IEMGMKSWDDVVAR....LKTIEGYQAAFE.KAFGK.........DAISKDNATKAIAAY
EGL01965 : IEMGMKSWDDVVAR....LKTIDGYQEAFA.AAFPG.........GEISKDTATKAIAAY
EFO07874 : IEMGMKNWDDVVTR....LQAIAGYQKAFE.EAFGK.........DSISKDNATKAIAAY
EEF80583 : NEMANPSFGVVIEK....LRNLEDYVGLFEAAFDGQ..........QASFETIPKAIASY
EEF81059 : NEMANPSFGVVIEK....LRNLEDYVGLFEAAFDGQ..........QASFETIPKAIASY
MCA0318 : NEMGNPSVGFVLDR....IESLPDYRGLFEQAFGRG...........PGMETVGQALACY
AEG02508 : NEMGNPSIGYVVDK....IKASADYRGWFEKAFNKG...........PTMETIGQALASY
EGL04739 : DEMANPSIGYVLET....VANSEDYRGLFQKAFGKG...........PGMETLGMAIASY
MCA0444 : VEHGGKGDFCEDEA....FANALGWSGDGSPGTGSL[25]LPTDEELTALEAYMLSLGRT
AEG01086 : ETIAAGGEFAEDED....FAHATGWSGDGAPGSGSL[25]LPSDAELDAIEAYLLSLGRS
ABA58262 : RTSIASPEGPRLGW....SGDGSPRDGTLKSFAIGA.[6]....KTLNRIPGVDFRLPTE
EGL03975 : VEMSCKGRSFADIG[20]LAGLRDPSGTGLTLTYDE[25]FYSQMEANFAMFFGLAIQQY
MCA2590 : QEMSCNGRTFPDVG[20]LAGYRSERGQGLAQTYAE[31]PYTLMEANFALYFGVAVQAY
AEG01090 : LEMTCANRSIADIG[20]FGTLNLTNSAEGNLQPGL[33]AYTQMEANFPMFFGLAIQMY
ABA76906 : VEMSCGGRTFADIG[20]LAPVSGARRPTYRELIKN[18]PYTQMEANFPLFFGLAIQMY


 * 200 * 220 * 240
MauG_Pn : QATGEFSPFDSKYDRVMRG.........EEKFT.PLEEFGYTVFIT...WNCRLCHMQRK : 231
MauG_Me : QRTLVSG..EAPFDRWALGD........ESAIG.ADAKRGFALFTG..KAGCAACHSTWR
MauG_Mf : QRTLVSK..LAPFDVWVEGD........ESAMS.ERAKRGFAVFKG..KARCATCHSSWR
MauG_Mm : ERTLVSN..AAPFDRWVAGD........ESAIS.ERSKHGFKLFTG..KAQCASCHKSWR
MauG_Mt : QRTLVSN..ISSFDRWVDGD........EQAIS.SSAQRGFDVFNG..KAQCAACHKSWR
AAR34912 : EGSTESNAFTSKYDAFLAG.........KAFLT.PEERRGLTLFNG..KAKCARCHVNTG
MCA1769 : EASREVSSFSSKYDAYLRG.........RAVLT.KQEKKGMALFEG..KAKCANCHSTRG
MCA1906 : ERSPVFARFDSKYDYYLKG.........KVKLT.PQEMRGLVIFESEEKGNCAACHPSRP
EGL02226 : ERSPVLNRFSSKYDYYLFG.........RAAFT.AQERRGLTVFEAGNKGNCAACHPNRP
AEG01769 : EQTAVFNKFNSKFDYVLAG.........KTRFT.PLEAQGFEVFNREDKGNCAACHVSEA
CcpA_Pa : EATLVTP..DSPFDLYLKGD........DKALD.AQQKKGLKAFM...DSGCSACHNGIN
Ccp_Pp : EATLITP..NSAFDRFLAGD........DAAMT.DQEKRGLQAFM...ETGCTACHYGVN
CcpA_Rc : EATLITP..NSKFDQWLMGA........DGAMS.ADEKAGLKLFI...DTGCAACHNGIN
Ccp_Mh : EATLITP..EAPFDKYLRGD........TSALN.ESEKEGLALFM...DRGCTACHSGVN
Ccp_Ne : EETLVTP..GSKFDKWLEGD........KNALN.QDELEGYNLFK...GSGCVQCHNGPA
CcpA_Gs : EATLITP..DSPFDQYLKGK........KKALD.GKQTAGLKLFL...DKGCVACHGGLN
MCA0345 : ERTLITP..NSPYDRYVGGD........KTALT.EQQVRGMNTFA...ETGCSNCHSGPA
AEG01939 : ERTLITP..NSAYDKYVKGD........KSAMN.EQQVRGMNKAV...ELGCTSCHSGPA
EGL01965 : ERTLITP..NSAYDKYANGD........QSALT.EQQVRGLNKAA...ELGCTSCHSGPA
EFO07874 : ERTLITP..NSPYDKYVSGD........KSALS.AQQVRGMEKVA...ELGCTTCHSGPA
EEF80583 : ERTLNSA..NSPFDRWYYGKE.......KDAMP.PSAIRGFDLFAG..KAQCIACHSVTE
EEF81059 : ERTLNSA..NSPFDRWYYGKE.......KDAMP.PSAIRGFDLFAG..KAQCIACHTVTE
MCA0318 : ERVLVSG..DSPFDRWRYGHE.......SGALS.EAARKGFELFTG..KAGCAACHTVGD
AEG02508 : QRTLNSA..DSPFDRWYFGKQ.......SQALS.DAAQRGFKLFTG..KAACSSCHSIGE
EGL04739 : ERTLNSA..DSPFDRWFYGKD.......RKVLS.EEAQQGFKLFTG..KAGCSGCHTVDS
MCA0444 : QDIDLSK.LSFNSPLVQQGKL.......LFDVK.ENPVRNGEVILG.ETANCNGCHQNAG
AEG01086 : KDYPVYQ.ISFHDPLVESGKV.......LFDTK.TNPVVDGEPAYG.QTANCNGCHQNAG
ABA58262 : DELIALE.AFQLSLGRQKDLHL......PLSLKGAVAKEGQKIFLDNSLGKCNICHQNAG
EGL03975 : ENTLIS..DDALFDREVDDDTGF.....PAGFT.EAQQRGFQVFN...DAHCNNCHSGPT
MCA2590 : MRTLIS..DQAPVDSDKVRVCFD[17]DAPQAMTAAQLRGLQHFI...NAHCIICHTGPL
AEG01090 : ESTLIS..DQAPIDTAVRDPDTY....KPVSLT.DSERRGMEVFT...ESHCNLCHAGPV
ABA76906 : EATLVS..DQAPIDAYLQGDH........TAMN.AQQVEGMNLFLG..KGKCVNCHGGPE
 * **

 * 260 * 280 * 300
MauG_Pn : QG......VAERETFTNFEYHNIGL.PVNE........TAREASGLGADHVDHGLLARPG : 276
MauG_Me : FT.........DDSF............................HDIGLKAGNDLGRGKFA
MauG_Mf : FT.........DDSF............................HDIGLPS.LDPGRGARV
MauG_Mm : FT.........DDSF............................HDIGLRT.EDIGRGAKV
MauG_Mt : FT.........DDSF............................HDIGLDS.PDQGRGKVI
AAR34912 : RA.....PLFTDYTYDNLGVPRNSENPFY..........ESAFNPLGINWIDQGLGGFLA
MCA1769 : MSYAGKFPLFTDFTYVNTGVPRNPENPFYQ........MPAEFNPLGADWVDPGLGGFLA
MCA1906 : SA.DGTPPLFTDHTYDNLGVPKNPNNPFYA........LPPTLNPDGPAFVDLGLGKRVG
EGL02226 : VN..GTPPLFTDHSYDNIGVPKNPENPFYG........LAPQFNPDGAYFVDLGLGGILD
AEG01769 : TV..AEDGSIEPPLFTDFSYDNIGL..............PRNVNIPGNPEPNPGLGGRTD
CcpA_Pa : LG.........GQAY..FPF....................GLVKKPDASVLPSGDKGRFA
Ccp_Pp : FG.........GQDY..HPF....................GLIAKPGAEVLPAGDTGRFE
CcpA_Rc : IG.........GNGY..YPF....................GVVEKPGAEVLPAGDKGRFA
Ccp_Mh : LG.........GQNY..YPF....................GLVAKPGAEILPEGDKGRFS
Ccp_Ne : VG.........GSSY.........................QKMGVFKPYETKNPAAGRMD
CcpA_Gs : LG.........GTGY..FPF....................GVVEKPAENILPLGDKGRFA
MCA0345 : FN..GPTLPEGTPFFMKFPT....................FENGMFEAKYGFSRDKGRAE
AEG01939 : FN........GPGMFQKFPM....................HPNGYFEAQYHFKKDKGLAE
EGL01965 : FN........GGGSFQKFPL....................HENGYFEAQYHFMKDKGVAE
EFO07874 : FN........GPGMFQKFPV....................TPNGYWEAQHHFSKDKGLAE
EEF80583 : KHALFTDNSLHNTGIGWERAMKKDPETQRVQVA.....PGRYLDVKNDIIKSVGSKKEGD
EEF81059 : KHALFTDNSLHNTGIGWERAMRKDPESQRVQVA.....PGRYVNVKNDIIKSVGNQQEGD
MCA0318 : RHALFTDDALHNTGVGYRASMDKTPAARRVQVA.....PGVSFEVDAKTFAQVAEPVPGD
AEG02508 : KTALFTDQKRHNTGIGYADSMQKAPEKQRVQVA.....PGVFVDVDNKSLQGVAEAKAND
EGL04739 : KFALFTDNGFHNTGIGFAAAMDGSNAKRRVQIA.....PGTFVEVDREVIDSVSGDKGND
MCA0444 : ANSSTTHANPTRDTGVENMRTP.................PAFLLDPALAVDGGFGKEERV
AEG01086 : GRSSSTKANPTRNTGVENMKIHP.................ARLLVPDMAYDGGFGVTESA
ABA58262 : ANARLGGQNVGN............................ANFDTGVENLPDSAMGGERR
EGL03975 : FSSAASPQIFLNTTKKPKYLKLVNRDVLGEQAS.[86]VCKGPGKKQARIPTPEIVAVEL
MCA2590 : ASTAVSPEIVRLVNGKAVKVNPNGYTLVDRTND.[95]CVLSKLAMIPTPAAAAAEVAKA
AEG01090 : MTTNAIVSNSLLVTPTPNAFFGPQHSLRAFGPE[133]SQNCQDPDYAYIPTVDAAIAAF
ABA76906 : LTNAASRLLMHPRERIERMVMADNLTTLYDNGF.[40]DPLDVDVCTFEAPLSAAIPCDA


 * 320 * 340 * 360
MauG_Pn : IED......PAQSGRFKVPSLRNV......AVTGPYMHNGVFTDLRTAILFYNKYTSRRP : 324
MauG_Me : PPSV.....TAMRYAFKTPSLRDL......RMEGPYMHDGQLGSLEAVLDHYIKGGEKRP
MauG_Mf : PPQV.....TIMQHAFKTPTLRDL......PRNGPFMHDGSMHSLDEVIRHYEQGGLQRP
MauG_Mm : PPQV.....TLMQYAFKTPSLRDL......PINGPYMHDGAMSGLDEVVKHYEKGGIDRK
MauG_Mt : PVEV.....TIMQHAFKTPTLRDL......PENGPFMHDGSMTDLEEVVKHYEEGGIQRA
AAR34912 : SRIDYSRFATANLGKHKVPTLRNVDKKTSPDFVKAFGHNGYFKSLKEIVHFYNTRDVLPT
MCA1769 : GRVEYAPYAADNKGKQKVPTLRNVDKRPSLAYLKAYMHNGAFKSLKEVVHFYNTRDVLAA
MCA1906 : K........FWEDGKLKVPTLRNI......ALTAPYMHNGYFSTLRSVVDFYNTRDVRPT
EGL02226 : A........PLEEGKIKVSTLRNV......AVTAPYTHNGYFKTLRGVVEFYSTRDLKPR
AEG01769 : LTTD....AADELGKHKVMSLRNI......AITAPYGHNGSMATLEQIVHFYNTRDTLGS
CcpA_Pa : VTKT.....QSDEYVFRAAPLRNV......ALTAPYFHSGQVWELKDAVAIMGNAQLG..
Ccp_Pp : VTRT.....TDDEYVFRAAPLRNV......ALTAPYFHSGVVWELAEAVKIMSSAQIG..
CcpA_Rc : VTAT.....ADDEYVFRAGPLRNI......ALTAPYFHSGKVWDLREAVSVMANSQLG..
Ccp_Mh : VTET.....ASDEYVFRASPLRNI......ELTAPYFHSGAVWSLEEAVAVMGTAQLG..
Ccp_Ne : VTGN.....EADRNVFKVPTLRNI......ELTYPYFHDGGAATLEQAVETMGRIQLN..
CcpA_Gs : VTNT.....AKDEYVFRAPSLRNV......AITYPYFHSGVVWSLKEAVAVMGSAQFG..
MCA0345 : VTKK.....TEDEHLFKVPTLRNV......ALTAPYFHNGKVKTLDEAVRVMAKLQLN..
AEG01939 : VTKK.....ADDEHMFKVPTLRNV......ALTAPYFHNGSVPTLDKAVKLMAKLQLG..
EGL01965 : VSKN.....PADEHLFKVPTLRNV......ALTAPYFHNGSVKTLDQAVKLMAKLQLD..
EFO07874 : VSKN.....AADEHLFKVPTLRNI......ALTAPYFHNGSVKTLDSAVTLMAKLQLG..
EEF80583 : LGHYEVTQDPADRWKYRTPSLRNV......ALTAPYMHDGSMPNLESVVEFYNNGGIENE
EEF81059 : LGHYEVTQDPADRWKYRTPSLRNV......ALTAPYMHDGSLGTLEEVVTFYNEGGIKNE
MCA0318 : LGRYEITQNPADRWKYRTPTLRNV......ALTAPYMHNGVFASLREVVEFYNRGGEPNE
AEG02508 : LGYYEISQNPTDRWAYKTPSLRNV......ALSAPYMHNGSLASLKQVVAFYNQGGVANE
EGL04739 : LGRYEITQKPEDRWKYRTLSLRNI......GLTAPYMHDGSLGTLEEVVQFYRQGGRPNE
MCA0444 : SCGFSQDHTCYGNGRFNTPPLVEA......ADTAPFFHNNSVNTLEEAIASYNGDSFNQS
AEG01086 : CGPHR.DQVCYGDGRFNTPPLIEA......ADTPPFFHNNAVSTLEEAIAAYNSEAFNAS
ABA58262 : PPDDGLG..TPGDGTFNTPPLVEA......ADTAPFFHNHVIDTLEGAVAFYNSKAFNNS
EGL03975 : AKSGQGRLSGGVGAAFKIPTLRNV......ELTGPYMHNGGMKSLEEVVEFYNRGGNLTN
MCA2590 : EAGSSSLLRAGVQGTFKIPTLRNV......ELTAPYFHNGSVLTLNQVIDFYIRVGNYAS
AEG01090 : NNPDDRRFLIGSKAAFKIPSLRNI......ELTGPYMHNGSMATLEQVIEFYARGGNFDN
ABA76906 : TLKPNVGFRDSVDGAFKTPTLRNI......ALTGPYFHNGSRSTLKQVMEFYNRGGDRRG
 * * *

 * 380 * 400 * 420
MauG_Pn : EAKINPET.............GAPWGEPEVARNLSLAELQSGLMLDDGRVDALVAFLET. : 370
MauG_Me : SLSFEMKP..................................FEMSERERRDLVAFLET.
MauG_Mf : SISAEMKR..................................FELTETEREYLIEFIHT.
MauG_Mm : SRSLEMKP..................................FELTDAERLSLIEFVKT.
MauG_Mt : SLSKEMKP..................................FELTDQQRADLIAFLKT.
AAR34912 : CAPGSPGEK.............VTCWPEPELALTMNTTELGNLKLSDAEEDALVAFMKT.
MCA1769 : CEHLSHPEP............GINCWPAAEEAANVNRTETGDLKLSDEEEDAIVAFLRT.
MCA1906 : CWNELVPEAD.........AQRLGCWPAPEVKQNVNGDELGNLRLSDREVDDLVAFLKT.
EGL02226 : CRNPLTTEAK.........ARAQKCWPAAEVIANVNHGELGSLRLTPREIGDLVAFLKT.
AEG01769 : VDDINHPG............FGKTGWPEPEFSENLNSDELGNLGLSDDEEKALVAFLKT.
CcpA_Pa : ..........................................KQLAPDDVENIVAFLHS.
Ccp_Pp : ..........................................TELTDQQAEDITAFLGT.
CcpA_Rc : ..........................................ATLDDTQVDQITAFLGT.
Ccp_Mh : ..........................................TELNDDEVKSIVAFLKT.
Ccp_Ne : ..........................................REFNKDEVSKIVAFLKT.
CcpA_Gs : ..........................................IKLSDDESEAIAAFLGS.
MCA0345 : ..........................................KDLSDQQVADVVAFLNA.
AEG01939 : ..........................................KDLSDEDTADIVAFLNA.
EGL01965 : ..........................................KDLPKQDVDDIVAFLNA.
EFO07874 : ..........................................KDLSKEEVADIVAFLNG.
EEF80583 : TQSPLINP..................................LNLTKNEMDDLVEFLKA.
EEF81059 : IQSPLIQP..................................LNLTSAEMEDLVAFLKS.
MCA0318 : NLDPLIRP..................................LGLSAQEVDALVEFLSS.
AEG02508 : NLSPLIKP..................................LGLSAAEIDDLVAFLQA.
EGL04739 : NLDPLIRP..................................LPLNDREAAALVAFLKS.
MCA0444 : PGAKTSSGKDRRVKL[19]NIRSSNSLSRKSMDLRGRNAKETLKLAMADTEDAIEVLQG.
AEG01086 : PGSLSKDKDRSVKLD[19]IRQSDKLDNQAKRISNNATARELAKLGRKENQDAIRVLKE.
ABA58262 : PAGQVLASGD...........PNGIGIQLNESQVAAVTVFLRVINALENIRWSIELIEG.
EGL03975 : PRHSTTLVFFQG................................MSAQDKSDLVAFLKT.
MCA2590 : RDKAVEMPTLS.............................DIIGNEQGPKDDIVAFLKS.
AEG01090 : SNQSDFLTRTP...............................MSDNAQKRADLLAFLKT.
ABA76906 : EDANNTSGFEHPAVN..............QHNTSNLDPDMTALNLTPDEIDALVKFMEVG


 * 440 *
MauG_Pn : LTDRRYEPLLEESRAAQKD............. : 389
MauG_Me : LKAEPAAITLPQLP..................
MauG_Mf : LDGGLLDIEPPQLPE.................
MauG_Mm : LDGGALKVDYPRMPQ.................
MauG_Mt : LDGGPLAIEPPVLPKE................
AAR34912 : LTDGYQP.........................
MCA1769 : LSDGFQLSGPTAD...................
MCA1906 : LTDGYRPE........................
EGL02226 : LTDGYRNGSPWQFKAP................
AEG01769 : LTDDYPKWGHDRRVPPWSPSPFAQTVRP[18]
CcpA_Pa : LSGKQPRVEYPLLPASTETTPRPAE.......
Ccp_Pp : LTGEQPVIDHPILPVRTGTTPLPTPM......
CcpA_Rc : LTGEQPEVVHPILPVRSAQTPRPEHMN.....
Ccp_Mh : LTGNVPEVTYPVLPPSTANTPKPVDMIP....
Ccp_Ne : LTGDQPDFKLPILPPSNNDTPRSQPYE.....
CcpA_Gs : LTGKQPKVVYPIMPASTDATPRPRL.......
MCA0345 : LTGEFPKQQMPQLPGLPNGTFDYD........
AEG01939 : LTGEFPKQTMPTLPGTPGSTFN..........
EGL01965 : LTGEFPKQTMPRLPGTPDKTFNYD........
EFO07874 : LTGEFPKQKMPVLPATLGSTFN..........
EEF80583 : LNGDNVTEIISDSFATPIGDHSNN........
EEF81059 : LNGDNVAEIISDSFATPIGDHSKN........
MCA0318 : LTGGDNQTLVSDAFAAPVGNGR..........
AEG02508 : LNGSNVETLVSDAFAAPVGDSQ..........
EGL04739 : LTGSNVGDLVGDAFAAPIGDAK..........
MCA0444 : GTLLPYPEALARLEEALALEKKALHTEI[27]
AEG01086 : GVLGTNWKAVQKLEKAEYYQRLALLAPV[44]
ABA58262 : AMGRKFAIFGDVDEQLRIAGFEIGDAVM[44]
EGL03975 : LTDERVRWERAPFDHPALQVPHGHEGGE[39]
MCA2590 : LTDERVRDEAAPFDHPALRVPNGHAGDH[48]
AEG01090 : LTDDRVRYEQAPFDHPELIVPNGHDGDD[39]
ABA76906 : LTDPRVAWERAPFDHPSLVIPQGHIGDE[46]

**Fig. S1:** Condensed multiple sequence alignment for known MauG proteins and bacterial di-haem cytochrome *c* peroxidase proteins to the homologues in *M. capsulatus* and their closest BLASTP hits. Protein codes are as follows: MauG from *Paracoccus* *denitrificans* (MauG_Pn), *Methylobacterium* *extorquens* (MauG_Me), *Methylobacillus* *flagellatus* (MauG_Mf), *Methylotenera* *mobilis* (MauG_Mm), *Methylophaga* *thiooxydans* (MauG_Mt); cytochrome *c* oxidase from *Pseudomonas* *aeruginosa* (CcpA_Pa), *Paracoccus* *pantotrophus* (Ccp_Pp), *Rhodobacter* *capsulatus* (CcpA_Rc), *Marinobacter* *hydrocarbonoclasticus* (Ccp_Mh), *Nitrosomonas* *europaea* (Ccp_Ne), *Geobacter* *sulfurreducens* (CcpA_Gs), the dihaem cytochrome *c* from *M. capsulatus* are given with their MCA-number and shaded in yellow. Homologues to the *M. capsulatus* BCCP/MauG dihaem family proteins are given with their Genebank accession number; *G*. *sulfurreducens* (AAR34912), *Methylomicrobium* *album* (EGL02226, EGL01965, EGL04739, EGL03975), *Methylomonas* *methanica* (AEG01769, AEG01939, AEG02508, AEG01086, AEG01090), *Methylobacter* *tundripaludum* (EFO07874), *M*. *thiooxydans* (EEF80583, EEF81059), *Nitrosococcus* *oceani* (ABA58262), *Pseudomonas* *fluorescens* (ABA76906). Alignment was done using ClustalX (v.2.0) and edited using the GeneDoc software. Residues that are physiochemical identical are shaded in black (100% conserved) and grey (60%< conserved). Number in brackets denotes residues omitted from the alignment for easier viewing. The CxxCH motifs that indicates the location of the *c*-type hemes are indicated with * shaded in red for the conserved residues. The axial ligand for the six-coordinated heme (Y314 for MauG_Pn) is indicated with * shaded in magenta, the tryptophan important for electron transport between the two *c*-type hemes (W113 for MauG_Pn) is indicated with * shaded in green, and residues important for calcium binding (N86, T295, and P297 in MauG_Pn) are indicated with * shaded in cyan.

References

Fülöp V., Ridout, C.J., Greenwood, C., and Hajdu, J. (1995) Crystal structure of the di-haem cytochrome *c* peroxidase from *Pseudomonas* *aeruginosa*. *Structure* **3**: 1225-1233.

Jensen, L.M.R., Sanishvili, R., Davidson, V.L., and Wilmot, C.M. (2010) In crystallo posttranslational modification within a MauG/pre-methylamine dehydrogenase complex. *Science* **327**: 1392-1394.

Shin, S., Feng, M., Chen, Y., Jensen, L.M., Tachikawa, H., Wilmot, C.M., Liu, A., and Davidson, V.L. (2011) The tightly bound calcium of MauG is required for tryptophan tryptophylquinone cofactor biosynthesis. *Biochemistry* **50**: 144-150.
