# Supplementary material for: Transcriptomic profiling of Methylococcus capsulatus (Bath) during growth with two different methane monooxygenases
Source: Microbiologyopen. 2015 Dec 20;5(2):254–67. doi: 10.1002/mbo3.324 (PMC4831470; doi:10.1002/mbo3.324)
Supplement: Supplementary file 2 — Figure S2. Alignment of MCA1187 to cytochrome bd subunit I and homologs. [file MBO3-5-254-s002.pptx]

## Slide 1
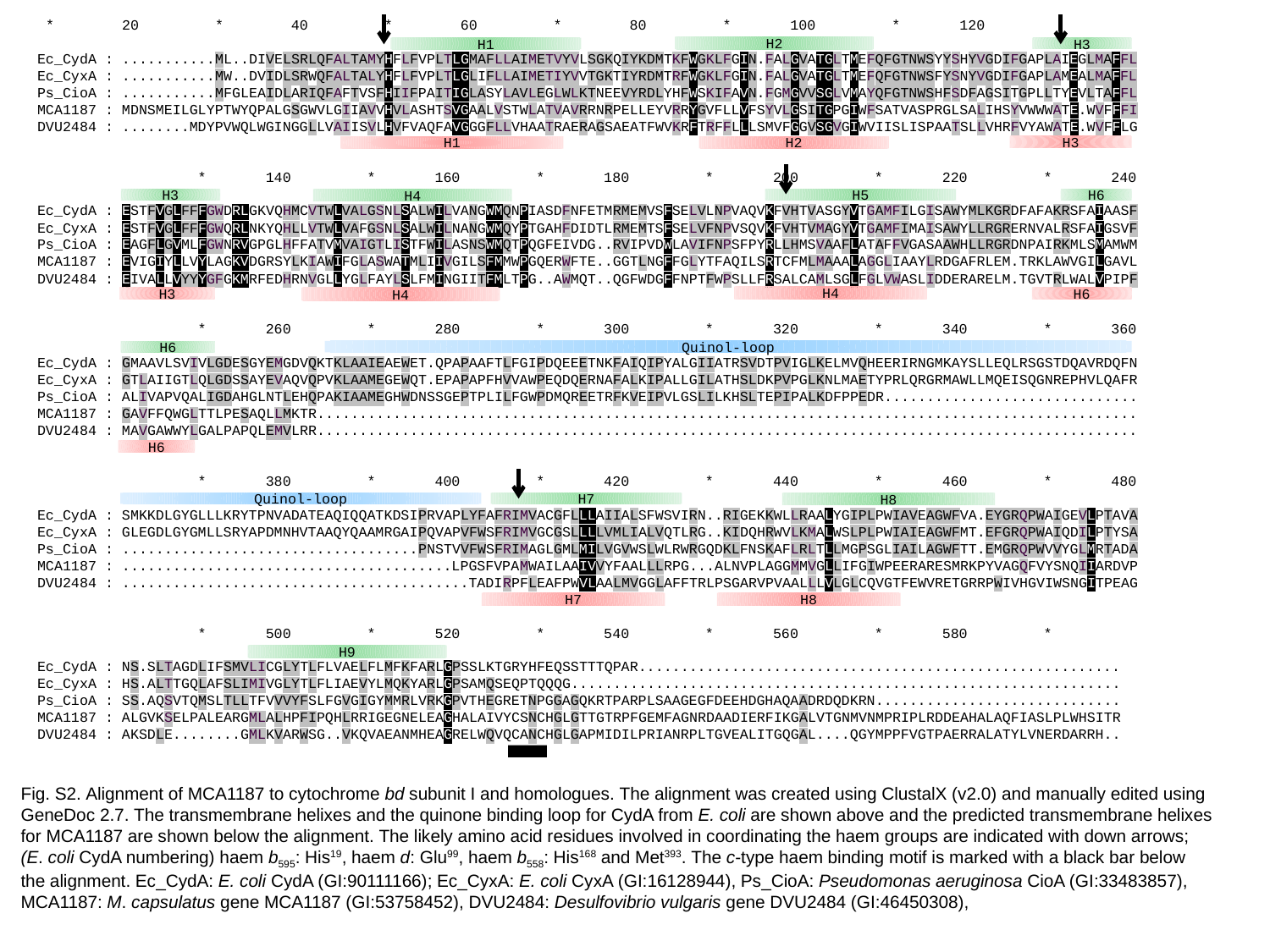

* 20 * 40 * 60 * 80 * 100 * 120
Ec_CydA : ...........ML..DIVELSRLQFALTAMYHFLFVPLTLGMAFLLAIMETVYVLSGKQIYKDMTKFWGKLFGIN.FALGVATGLTMEFQFGTNWSYYSHYVGDIFGAPLAIEGLMAFFL
Ec_CyxA : ...........MW..DVIDLSRWQFALTALYHFLFVPLTLGLIFLLAIMETIYVVTGKTIYRDMTRFWGKLFGIN.FALGVATGLTMEFQFGTNWSFYSNYVGDIFGAPLAMEALMAFFL
Ps_CioA : ...........MFGLEAIDLARIQFAFTVSFHIIFPAITIGLASYLAVLEGLWLKTNEEVYRDLYHFWSKIFAVN.FGMGVVSGLVMAYQFGTNWSHFSDFAGSITGPLLTYEVLTAFFL
MCA1187 : MDNSMEILGLYPTWYQPALGSGWVLGIIAVVHVLASHTSVGAALVSTWLATVAVRRNRPELLEYVRRYGVFLLVFSYVLGSITGPGIWFSATVASPRGLSALIHSYVWWWATE.WVFFFI
DVU2484 : ........MDYPVWQLWGINGGLLVAIISVLHVFVAQFAVGGGFLLVHAATRAERAGSAEATFWVKRFTRFFLLLSMVFGGVSGVGIWVIISLISPAATSLLVHRFVYAWATE.WVFFLG
 * 140 * 160 * 180 * 200 * 220 * 240
Ec_CydA : ESTFVGLFFFGWDRLGKVQHMCVTWLVALGSNLSALWILVANGWMQNPIASDFNFETMRMEMVSFSELVLNPVAQVKFVHTVASGYVTGAMFILGISAWYMLKGRDFAFAKRSFAIAASF
Ec_CyxA : ESTFVGLFFFGWQRLNKYQHLLVTWLVAFGSNLSALWILNANGWMQYPTGAHFDIDTLRMEMTSFSELVFNPVSQVKFVHTVMAGYVTGAMFIMAISAWYLLRGRERNVALRSFAIGSVF
Ps_CioA : EAGFLGVMLFGWNRVGPGLHFFATVMVAIGTLISTFWILASNSWMQTPQGFEIVDG..RVIPVDWLAVIFNPSFPYRLLHMSVAAFLATAFFVGASAAWHLLRGRDNPAIRKMLSMAMWM
MCA1187 : EVIGIYLLVYLAGKVDGRSYLKIAWIFGLASWATMLIIVGILSFMMWPGQERWFTE..GGTLNGFFGLYTFAQILSRTCFMLMAAALAGGLIAAYLRDGAFRLEM.TRKLAWVGILGAVL
DVU2484 : EIVALLVYYYGFGKMRFEDHRNVGLLYGLFAYLSLFMINGIITFMLTPG..AWMQT..QGFWDGFFNPTFWPSLLFRSALCAMLSGLFGLVWASLIDDERARELM.TGVTRLWALVPIPF
 * 260 * 280 * 300 * 320 * 340 * 360
Ec_CydA : GMAAVLSVIVLGDESGYEMGDVQKTKLAAIEAEWET.QPAPAAFTLFGIPDQEEETNKFAIQIPYALGIIATRSVDTPVIGLKELMVQHEERIRNGMKAYSLLEQLRSGSTDQAVRDQFN
Ec_CyxA : GTLAIIGTLQLGDSSAYEVAQVQPVKLAAMEGEWQT.EPAPAPFHVVAWPEQDQERNAFALKIPALLGILATHSLDKPVPGLKNLMAETYPRLQRGRMAWLLMQEISQGNREPHVLQAFR
Ps_CioA : ALIVAPVQALIGDAHGLNTLEHQPAKIAAMEGHWDNSSGEPTPLILFGWPDMQREETRFKVEIPVLGSLILKHSLTEPIPALKDFPPEDR..............................
MCA1187 : GAVFFQWGLTTLPESAQLLMKTR.................................................................................................
DVU2484 : MAVGAWWYLGALPAPQLEMVLRR.................................................................................................
 * 380 * 400 * 420 * 440 * 460 * 480
Ec_CydA : SMKKDLGYGLLLKRYTPNVADATEAQIQQATKDSIPRVAPLYFAFRIMVACGFLLLAIIALSFWSVIRN..RIGEKKWLLRAALYGIPLPWIAVEAGWFVA.EYGRQPWAIGEVLPTAVA
Ec_CyxA : GLEGDLGYGMLLSRYAPDMNHVTAAQYQAAMRGAIPQVAPVFWSFRIMVGCGSLLLLVMLIALVQTLRG..KIDQHRWVLKMALWSLPLPWIAIEAGWFMT.EFGRQPWAIQDILPTYSA
Ps_CioA : ...................................PNSTVVFWSFRIMAGLGMLMILVGVWSLWLRWRGQDKLFNSKAFLRLTLLMGPSGLIAILAGWFTT.EMGRQPWVVYGLMRTADA
MCA1187 : .......................................LPGSFVPAMWAILAAIVVYFAALLLRPG...ALNVPLAGGMMVGLLIFGIWPEERARESMRKPYVAGQFVYSNQIIARDVP
DVU2484 : .........................................TADIRPFLEAFPWVLAALMVGGLAFFTRLPSGARVPVAALLLVLGLCQVGTFEWVRETGRRPWIVHGVIWSNGITPEAG
 * 500 * 520 * 540 * 560 * 580 *
Ec_CydA : NS.SLTAGDLIFSMVLICGLYTLFLVAELFLMFKFARLGPSSLKTGRYHFEQSSTTTQPAR.........................................................
Ec_CyxA : HS.ALTTGQLAFSLIMIVGLYTLFLIAEVYLMQKYARLGPSAMQSEQPTQQQG.................................................................
Ps_CioA : SS.AQSVTQMSLTLLTFVVVYFSLFGVGIGYMMRLVRKGPVTHEGRETNPGGAGQKRTPARPLSAAGEGFDEEHDGHAQAADRDQDKRN.............................
MCA1187 : ALGVKSELPALEARGMLALHPFIPQHLRRIGEGNELEAGHALAIVYCSNCHGLGTTGTRPFGEMFAGNRDAADIERFIKGALVTGNMVNMPRIPLRDDEAHALAQFIASLPLWHSITR
DVU2484 : AKSDLE........GMLKVARWSG..VKQVAEANMHEAGRELWQVQCANCHGLGAPMIDILPRIANRPLTGVEALITGQGAL....QGYMPPFVGTPAERRALATYLVNERDARRH..
H2
H3
H1
H3
H2
H1
H3
H4
H6
H5
H4
H6
H3
H4
Quinol-loop
H6
H6
H7
Quinol-loop
H8
H8
H7
H9
Fig. S2. Alignment of MCA1187 to cytochrome bd subunit I and homologues. The alignment was created using ClustalX (v2.0) and manually edited using
GeneDoc 2.7. The transmembrane helixes and the quinone binding loop for CydA from E. coli are shown above and the predicted transmembrane helixes
for MCA1187 are shown below the alignment. The likely amino acid residues involved in coordinating the haem groups are indicated with down arrows;
(E. coli CydA numbering) haem b595: His19, haem d: Glu99, haem b558: His168 and Met393. The c-type haem binding motif is marked with a black bar below
the alignment. Ec_CydA: E. coli CydA (GI:90111166); Ec_CyxA: E. coli CyxA (GI:16128944), Ps_CioA: Pseudomonas aeruginosa CioA (GI:33483857),
MCA1187: M. capsulatus gene MCA1187 (GI:53758452), DVU2484: Desulfovibrio vulgaris gene DVU2484 (GI:46450308),
