# Supplementary material for: Transcriptomic profiling of Methylococcus capsulatus (Bath) during growth with two different methane monooxygenases
Source: Microbiologyopen. 2015 Dec 20;5(2):254–67. doi: 10.1002/mbo3.324 (PMC4831470; doi:10.1002/mbo3.324)
Supplement: Supplementary file 3 — Table S1. List of differentially expressed genes between cells producing sMMO and pMMO. Table S2. Summary of genes encoding c‐type cytochromes in M. capsulatus. Table S3. List of primers used in qRT‐PCR. Table S4. List of unassigned peptides from the LTQ‐ORBITRAP mass spectrometry analyses of the M. capsulatus cellular ffractions. [file MBO3-5-254-s003.zip › mbo3324-sup-0004-TableS2.doc]

Table S2. Summary of genes encoding *c*-type cytochromes in *M*. *capsulatus* strain Bath.

| Locus Tag | Annotation / function | Mol. Mass^a^ | Signal peptide^b^ | Detected by MS^c^ |
| --- | --- | --- | --- | --- |
| MCA0179b | New. Putative membrane embedded *c*-type cytochrome | 29.0 (1) | NP, TMH | ND |
| MCA0318 | MauG family protein with an N-terminal thioredoxin-like region | 66.6 (2) | 1 - 26 | ND |
| MCA0338 | Cytochrome *c*_553O_ family protein | 103.5 (9) | 1 - 29 | P |
| MCA0345 | Cytochrome *c* peroxidase | 35.8 (2) | 1 - 21 | S, P |
| MCA0421 | Cytochrome *c*_553O_ family protein | 118.9 (9) | 1 - 33 | S |
| MCA0423 | Occ. Cytochrome *c*_553O_ | 124.5 (8) | 1 - 33 | S |
| MCA0424 | Novel multihaem cytochrome *c* family protein | 101.6 (7*) | 1 - 26 | S |
| MCA0426 | Novel multihaem cytochrome *c* family protein | 100.1 (7*) | 1 - 27 | S |
| MCA0428 | New codon start. Cytochrome *c*_3_ family protein | 25.6 (7) | 1 - 20 | ND |
| MCA0428b | New. Cytochrome *c*_3_ family protein. | 38.6 (8) | 1 - 25 | ND |
| MCA0429 | New. Multihaem *c*-type cytochrome | 35.5 (6) | 1 - 30 | ND |
| MCA0431 | New codon start. Multihaem *c*-type cytochrome. C-terminal show high sequence similarity to alpha subunit of assimilatory sulphite reductase | 109.7 (6*) | 1 - 32, TMH | ND |
| MCA0432 | Multihaem *c*-type cytochrome | 29.1 (3) | 1 - 23 | ND |
| MCA0433 | Multihaem *c*-type cytochrome | 106.9 (14) | 1 - 29 | ND |
| MCA0444 | MauG family protein | 74.0 (2) | 1 - 20 | S, OM^d^ |
| MCA0524 | CytL. Cytochrome P460 | 16.2 (1) | 1 - 17 | S, P |
| MCA0781 | MxaG. Electron acceptor for methanol dehydrogenase | 15.3 (1) | 1 - 25 | S, P, OM |
| MCA0879 | CoxB. Subunit II of terminal cytochrome *c* oxidase | 39.7 (1) | 1 - 21, TMH | IM^e^ |
| MCA0940 | Cytochrome *c*_4_ family protein. | 12.3 (1) | 1 - 24 | P |
| MCA0948 | Cytochrome *c*_5_ family protein | 12.3 (1) | 1 - 17 | ND |
| MCA0956 | HaoA. Hydroxylamine oxidoreductase | 62.0 (8) | 1 - 25 | IM |
| MCA1068 | Dihaem cytochrome *c*_4_ family protein | 20.5 (2) | 1 - 25 | S, P, IM |
| MCA1185 | Dihaem cytochrome *c*_4_ family protein | 22.3 (2) | 1 - 25, TatP | IM, OM^d^ |
| MCA1187 | Membrane embedded *c*-type cytochrome | 50.7 (1) | NP, TMH | IM, OM^d^ |
| MCA1493 | New codon start. Predicted carbohydrate-binding *c*-type cytochrome | 127.5 (2) | 1 - 31 | ND |
| MCA1505 | SoxD family cytochrome | 16.9 (1) | 1 - 21 | ND |
| MCA1585 | Cti. Fatty acid cis-trans isomerase | 88.0 (1) | 1 - 20 | P |
| MCA1769 | MauG family protein | 43.9 (2) | 1 - 26 | ND |
| MCA1775 | Class I *c*-type cytochrome | 28.3 (1) | 1 - 21 | P |
| MCA1787 | Putative membrane embedded *c*-type cytochrome | 28.4 (1) | NP, TMH | ND |
| MCA1799 | Cytochrome *c*_4_ family protein | 9.1 (1) | 1 - 22 | ND |
| MCA1806 | Cti. Fatty acid cis-trans isomerase | 93.3 (1) | NP, TatP? | ND |
| MCA1906 | MauG family protein | 42.2 (2) | 1 - 20 | S |
| MCA1960 | PetC. Cytochrome *c*_1_ subunit of ubiquinol-cytochrome *c* reductase | 25.6 (1) | 1 - 18, TMH | ND |
| MCA2010 | Predicted *c*-type cytochrome | 62.5 (1*) | 1 - 28 | ND |
| MCA2160 | Cytochrome *c*_553O_ family protein | 116.0 (9) | 1 - 36 | ND |
| MCA2181 | Cytochrome *c*_5_ family protein | 27.9 (1) | 1 - 20 | P |
| MCA2185 | Cytochrome *c*_5_ family protein | 12.0 (1) | 1 - 25 | ND |
| MCA2189 | Multihaem *c*-type cytochrome | 69.3 (21) | 1 - 29 | ND |
| MCA2190 | New. Multihaem *c*-type cytochrome | 37.1 (11) | 1 - 32 | ND |
| MCA2196 | Novel class I *c*-type cytochrome | 39.2 (1) | 1 - 38 | ND |
| MCA2197 | Novel class I *c*-type cytochrome | 36.5 (1) | 1 - 34 | ND |
| MCA2198 | Novel class I *c*-type cytochrome | 37.5 (1) | 1 - 42 | ND |
| MCA2259 | Cytochrome *c*_553O_ family protein | 115.0 (9) | 1 - 36 | S |
| MCA2394 | CytS. Cytochrome *c*’-β | 16.0 (1) | 1 - 20 | ND |
| MCA2395 | Dihaem cytochrome *c*_4_ family protein | 21.4 (2) | 1 - 19 | ND |
| MCA2401 | NorC. Cytochrome *c* subunit of nitric oxide reductase | 22.8 (1) | NP, TMH | ND |
| MCA2405 | Cytochrome *c*_551/552_ family protein | 9.4 (1) | 1 - 24 | P |
| MCA2590 | MauG family protein | 79.3 (2) | 1 - 41 | S |
| MCA2603 | Dihaem cytochrome *c*_4_ family protein | 20.3 (2) | 1 - 23 | P, IM |
| MCA2618 | Cytochrome *c*_555_ | 11.1 (1) | 1 - 27 | S, P |

a: molecular mass given in kDa calculated from the processed peptide and with potential heme groups (0.62 kDa per heme). Number of haem groups bound to CxxCH motifs per peptide is given in parentheses. ORFS with putative additional haem groups bound to Cx_n_CH (where n = 3 to 7) motifs are indicated with an *.

b: The length of the signal peptide is given as predicted by SignalP; TatP: predicted to be transported by the twin arginine transport system by TatP; TMH: predicted to have transmembrane helixes by TMHMM; NP: not predicted to have a signal peptide.

c: Peptides were analysed with LTQ-ORBITRAP. Only proteins that have peptides that gave scores > 20 are listed. S: surface fraction; P: periplasma fraction; IM: inner membrane fraction; OM: washed outer membrane fraction; ND: not detected.

d: only detected in sMMO expressing cells.

e: only detected in pMMO expressing cells.
